# Amygdala subnuclear volumes in temporal lobe epilepsy with hippocampal sclerosis and in non-lesional patients

**DOI:** 10.1093/braincomms/fcac225

**Published:** 2022-09-06

**Authors:** Alice Ballerini, Manuela Tondelli, Francesca Talami, Maria Angela Molinari, Elisa Micalizzi, Giada Giovannini, Giulia Turchi, Marcella Malagoli, Maurilio Genovese, Stefano Meletti, Anna Elisabetta Vaudano

**Affiliations:** Department of Biomedical, Metabolic and Neural Sciences, University of Modena and Reggio Emilia, Modena 41125, Italy; Azienda USL, Modena 41121, Italy; Department of Biomedical, Metabolic and Neural Sciences, University of Modena and Reggio Emilia, Modena 41125, Italy; Neurology Unit, OCB Hospital, AOU Modena, Modena 41126, Italy; PhD Program in Clinical and Experimental Medicine, University of Modena and Reggio Emilia, Modena 41121, Italy; Neurology Unit, OCB Hospital, AOU Modena, Modena 41126, Italy; PhD Program in Clinical and Experimental Medicine, University of Modena and Reggio Emilia, Modena 41121, Italy; Neurology Unit, OCB Hospital, AOU Modena, Modena 41126, Italy; Neuroradiology Unit, OCB Hospital, AOU Modena, Modena 41126, Italy; Neuroradiology Unit, OCB Hospital, AOU Modena, Modena 41126, Italy; Department of Biomedical, Metabolic and Neural Sciences, University of Modena and Reggio Emilia, Modena 41125, Italy; Neurology Unit, OCB Hospital, AOU Modena, Modena 41126, Italy; Department of Biomedical, Metabolic and Neural Sciences, University of Modena and Reggio Emilia, Modena 41125, Italy; Neurology Unit, OCB Hospital, AOU Modena, Modena 41126, Italy

**Keywords:** temporal lobe epilepsy, amygdala, hippocampal sclerosis, morphometric analyses, MRI

## Abstract

Together with hippocampus, the amygdala is important in the epileptogenic network of patients with temporal lobe epilepsy. Recently, an increase in amygdala volumes (i.e. amygdala enlargement) has been proposed as morphological biomarker of a subtype of temporal lobe epilepsy patients without MRI abnormalities, although other data suggest that this finding might be unspecific and not exclusive to temporal lobe epilepsy. In these studies, the amygdala is treated as a single entity, while instead it is composed of different nuclei, each with peculiar function and connection. By adopting a recently developed methodology of amygdala’s subnuclei parcellation based of high-resolution T_1_-weighted image, this study aims to map specific amygdalar subnuclei participation in temporal lobe epilepsy due to hippocampal sclerosis (*n* = 24) and non-lesional temporal lobe epilepsy (*n* = 24) with respect to patients with focal extratemporal lobe epilepsies (*n* = 20) and healthy controls (*n* = 30). The volumes of amygdala subnuclei were compared between groups adopting multivariate analyses of covariance and correlated with clinical variables. Additionally, a logistic regression analysis on the nuclei resulting statistically different across groups was performed. Compared with other populations, temporal lobe epilepsy with hippocampal sclerosis showed a significant atrophy of the whole amygdala (*p*_Bonferroni_ = 0.040), particularly the basolateral complex (*p*_Bonferroni_ = 0.033), while the non-lesional temporal lobe epilepsy group demonstrated an isolated hypertrophy of the medial nucleus (*p*_Bonferroni_ = 0.012). In both scenarios, the involved amygdala was ipsilateral to the epileptic focus. The medial nucleus demonstrated a volume increase even in extratemporal lobe epilepsies although contralateral to the seizure onset hemisphere (*p*_Bonferroni_ = 0.037). Non-lesional patients with psychiatric comorbidities showed a larger ipsilateral lateral nucleus compared with those without psychiatric disorders. This exploratory study corroborates the involvement of the amygdala in temporal lobe epilepsy, particularly in mesial temporal lobe epilepsy and suggests a different amygdala subnuclei engagement depending on the aetiology and lateralization of epilepsy. Furthermore, the logistic regression analysis indicated that the basolateral complex and the medial nucleus of amygdala can be helpful to differentiate temporal lobe epilepsy with hippocampal sclerosis and with MRI negative, respectively, versus controls with a consequent potential clinical yield. Finally, the present results contribute to the literature about the amygdala enlargement in temporal lobe epilepsy, suggesting that the increased volume of amygdala can be regarded as epilepsy-related structural changes common across different syndromes whose meaning should be clarified.

## Teaser

Ballerini *et al*. reported the volumetric differences of amygdala’s subnuclei in temporal epilepsies compared with controls and extratemporal epilepsies. The findings highlight an overall atrophy of the amygdala, particularly the basolateral portion, in patients with hippocampal sclerosis and a novel isolated hypertrophy of the medial amygdala in non-lesional temporal epilepsies.

## Graphical Abstract

**Graphical abstract:**
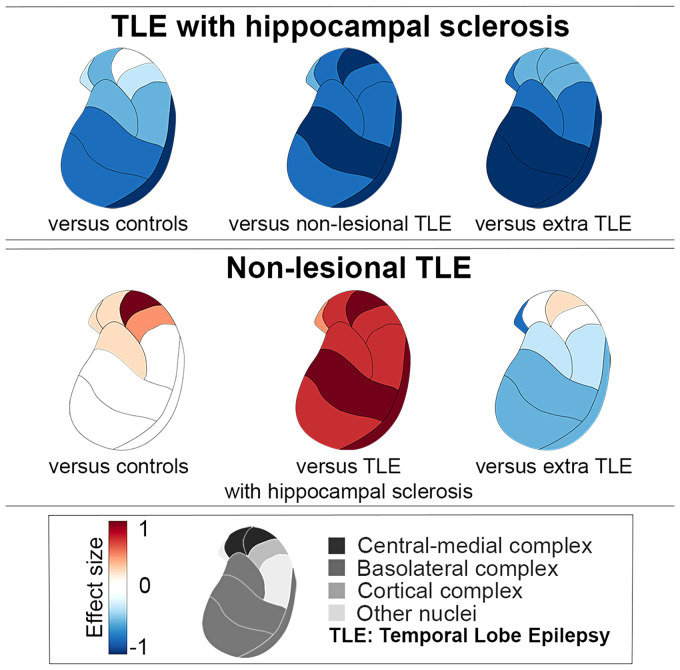

## Introduction

The amygdalar nuclear complex and hippocampal/parahippocampal region are key components of the limbic system that play a critical role in emotion, learning and memory, and complex behaviour.^1^ In temporal lobe epilepsy (TLE), the greatest attention has been focused on the hippocampus as hippocampal sclerosis (HS) is recognized as the most common cause of TLE.^2,3^ However, accumulating evidence suggests the amygdala as a key component in TLE in association or independent from HS.^4^ In patients with MRI-negative TLE (TLE-MRIneg), the absence of obvious epileptogenic lesions on routine visual assessment carries delays in surgical referral and in many cases the need for intracranial recordings before surgery. Advanced MRI morphometric approaches might contribute to reveal subtle structural abnormalities linked to the epileptogenic process.^5^ Recently, different studies described an increased amygdala volume (named amygdala enlargement, AE) in patients with TLE-MRIneg. Evaluation of AE differed between studies: while in some studies the increased amygdala’s volume was observed by qualitative visual assessment,^6-9^ in others it was revealed after post-processing MRI approaches.^3,10,11^ Overall, AE is reported on MRI in patients with non-lesional TLE at rates that range from 12 to 63%,^8,10,12^ leading to the hypothesis that AE represents a distinct subtype of TLE^6^ with specific nosological characteristics. This scenario however is complicated by the observation of AE also in patients with MRI-negative extra-TLE, thus suggesting that AE can be a feature associated to ‘non-lesional’ focal epilepsy^11^ but not specific to TLE.

The amygdala formation is commonly treated as a single entity in structural MRI; however, it is composed of multiple nuclei, each exhibiting different connectivity and histochemical profiles.^13^ Due to the small size of the amygdala, no prior studies focused on changes of amygdala subnuclei in patients with TLE. Thanks to recent advances in parcellation methods, it is possible to label amygdala subnuclei and automatically provide volumetric information for each one based on an in vivo atlas.^14^ The amygdala subnuclei might be further organized in groups or complexes based on their reciprocal connections and specific functions.^15^ These approaches have been successfully applied in patients with psychiatric conditions^16-18^ but up to date not in the epilepsy field.

In the present work, by investigating the morphometric characteristics of the amygdala substructures, we aim to map specific amygdala subnuclei participation in TLE-HS and TLE-MRIneg thus providing increased knowledge about the pathophysiological networks that mediate the amygdalar involvement in temporal lobe epilepsies.

## Materials and methods

### Study population

We retrospectively reviewed a cohort of consecutive patients with diagnosis of TLE who underwent a structural brain MRI study for diagnostic purposes at a 3 T MRI scan between April 2016 and April 2021 at the Neurology Unit, OCB Hospital (Modena, Italy).

The inclusion criteria were as follows: (i) aged older than 18 years and (ii) a brain MRI protocol encompassing at least a three-dimensional (3D) high-resolution T_1_-weighted (T1-3D) sequence.

We excluded patients with (i) abnormalities on the MRI scan except for HS; (ii) patients older than 65 years old; (iii) patients with progressive diseases (e.g. neurodegenerative disorders, encephalopathies); (iv) patients with previous neurosurgery; (v) patients in whom the diagnostic work-up (including cerebrospinal fluid analysis) suggested an autoimmune aetiology; (vi) patients with bilateral seizures’ onset zone based on clinical investigations; and (vii) patients with reported seizures in the 48 h before the MRI scan. This latter criterion is motivated by the intention to avoid any bias in amygdala volume estimation temporally related to the occurrence of ictal activity.^6,19^

TLE patients were divided in TLE-MRIneg, if no focal lesion was observed on the MRI, and TLE-HS if the structural MRI scan showed an alteration consistent with HS. A population of patients with focal epilepsy rather than TLE (extra-TLE) was included as an epilepsy control population. Inclusion and exclusion criteria were the same as TLE groups, except for the presence of focal cortical dysplasia (FCD) on the clinical MRI scan after expert evaluation.

All the recruited patients underwent a comprehensive diagnostic evaluation protocol which included the clinical history with seizures’ semiology, neurological examination, prolonged scalp video-EEG monitoring, and structural MRI scan. Interictal FDG-PET was performed when indicated. Epilepsy patients’ classification in this study was determined by board-certified neurologists (S.M., G.G., G.T., E.M., and A.E.V.) with expertise in epileptology and in accordance with criteria defined by the International League Against Epilepsy.^20,21^ Specifically, a diagnosis of TLE was performed in presence of at least one Video-EEG recorded seizure arising from the temporal lobe. Neuroradiological diagnosis and classification of patients were done on visual inspection by two neuroradiologists (M.M. and M.G.) with experience in epilepsy. In case of discordance, the final classification was reached after a thorough discussion with a neurologist (S.M. and A.E.V.). From each patient recruited, we collected clinical information regarding gender, age, handedness, side of the epileptic focus, age of seizure onset, duration of epilepsy, the drug response to antiseizures medications (ASMs), and type of ASM at the time of MRI scan. A patient was defined as drug-responder if she/he had sustained seizure-freedom during the last 12 months before the MRI scan.^22^ Psychiatric comorbidity was defined as a history of documented psychiatric and/or psychological therapy and/or previous psychiatric hospitalization.

Finally, the volume measurements of subcortical and amygdala nuclei in all the patients’ groups were compared with MRI data collected from 30 healthy controls (HCs) matched in age and gender studied with the same MRI protocol and analysis.

### MRI data and segmentation protocol

MRI was performed on two different 3T scanners adopting an epilepsy-dedicated protocol: a 3.0 T Philips Intera MRI scanner (Best, The Netherlands) (for patients recruited between 2016 and 2017), and a 3.0 T GE Healthcare MRI scanner (Chicago, USA) (for patients recruited after 2018). As common sequences, the protocols included a 3D T_1_-weighted sequence, a 3D fluid-attenuated inversion recovery (FLAIR), and a bidimensional coronal T_2_-weighted image acquired perpendicular to the long axis of the hippocampus. Details of the MRI sequences for each scanner are summarized in Supplementary Table 1. Of note, patients (both TLE and extra-TLE) with an increased signal on T_2_-weighted images on the amygdala, mono or bilaterally, were excluded from further analysis, after expert visual evaluation. This procedure was applied as changes in the amygdala signal at MRI (particularly increased in T2/FLAIR signal) might be secondary to recurrent seizures instead of reflecting structural modifications.^4,23^ T_1_-weighted images were analyzed using a standardized image toolbox (FreeSurfer, version 6.0, https://surfer.nmr.mgh.harvard.edu), quality assurance [outlier detection based on interquartile of 1.5 standard deviations (SDs) along with visual inspection of segmentation], and statistical methods. Visual inspections of subcortical segmentations were conducted following standardized ENIGMA protocols (http://enigma.usc.edu), used in prior genetic studies of brain structure,^24,25^ large-scale case–control studies of epilepsy^26,27^ and neuropsychiatric illnesses.^28,29^

**Figure 1:**
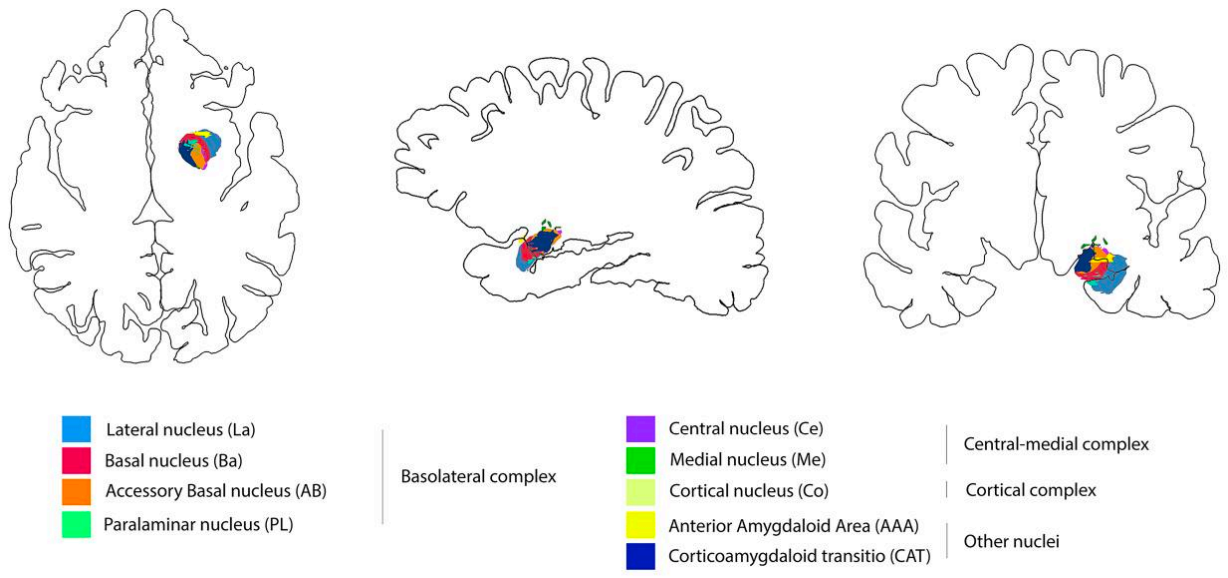
**Amygdala substructures.** Amygdala subnuclei segmentation module based on Saygin and Kliemann’s pipeline.^14^

The amygdala subnuclei segmentation module, which is only present in the FreeSurfer dev version (ftp://surfer.nmr.mgh.harvard.edu/pub/dist/freesurfer/dev), was used to parcellate the amygdala in nine nuclei for each side: anterior amygdaloid area (AAA), corticoamygdaloid transition area (CAT), basal nucleus (Ba), lateral nucleus (La), accessory basal nucleus (AB), central nucleus (Ce), cortical nucleus (Co), medial nucleus (Me), and paralaminar nucleus (PL) nuclei^14^ (Fig. 1). To account for correct subfield delineations, segmentations were visually inspected after processing. Analysts (A.B., M.T., and F.T.) were blind to participant diagnoses. Moreover, based on their cytoarchitectonics, histochemistry, and connections,^30^ the different nuclei of amygdala were subdivided into three main regions or complexes: (i) the deep group represented by the basolateral complex (BLA), which includes the lateral nucleus, the basal nucleus, the AB, and the PL; (ii) the superficial group named cortical complex (CC), which include the cortical nucleus; (iii) and the central–medial complex (CMC) composed by the medial and the central nuclei (Fig. 1).

In separate analyses, we also performed the segmentation of hippocampal subfields^31^ and thalamic structures^32^ as implemented in the FreeSurfer dev version (ftp://surfer.nmr.mgh.harvard.edu/pub/dist/freesurfer/dev). These additional segmentations were required because of the strict anatomical and functional relationships between the hippocampus, thalamus, and amygdala. As far as the hippocampal subfields we obtained the volumes of the following structures bilaterally: hippocampal body, hippocampal head, hippocampal tail, hippocampal fissure, subiculum, presubiculum, parasubiculum, CA1, CA2/3, CA4, molecular layer, granule cell and molecular layer of the dentate gyrus (GC-ML-GD), fimbria, and hippocampal-amygdala transition area (HATA). We also calculated the volumes of 25 individual thalamic nuclei for each side, including the anteroventral nuclei in the anterior group; the laterodorsal and lateral posterior nuclei in the lateral group; the ventral anterior, ventral anterior magnocellular, ventral lateral anterior, ventral lateral posterior, ventromedial, and ventral posterolateral nuclei in the ventral group; the central medial, central lateral, paracentral, centromedian, and parafascicular nuclei in the intralaminar group; the paratenial, medial ventral, mediodorsal medial magnocellular, and mediodorsal lateral parvocellular nuclei in the medial group; and the lateral geniculate, medial geniculate, suprageniculate, pulvinar anterior, pulvinar inferior, pulvinar lateral, and pulvinar medial nuclei in the posterior group.

### Statistical analysis

One-way ANOVAs were used to assess differences in demographic and clinical variables among groups when distributed normally, Kruskal–Wallis tests were performed otherwise. Fisher’s exact tests were performed on categorical variables. Volume measurements across the two different MRI scanners were harmonized using the ‘neuroCombat’^33,34^ package for R (https://cran.r-project.org/). After harmonization, the volumes of all subcortical structures and the volumes of amygdala subnuclei and complexes were converted into *z*-scores based on the mean and SD of HC population. To confirm the success of the scanner harmonization, we performed an independent sample *t*-test between the *z*-scored volumes of the whole left and right amygdala obtained after ComBat harmonization in all patients’ groups (Supplementary Table 2). The statistical significance of differences in mean volumes between left and right amygdala substructures in HC population was assessed using paired *t*-tests to check for asymmetries. To account for the side of the epileptic focus, subcortical measurements of right TLE and extra-TLE patients were flipped in order to have all the morphometric data of the epileptic focus on the left hemisphere. All morphometric subcortical analyses are then reported as ipsilateral or contralateral respect with the epilepsy focus. After testing the normality of morphometric data with Shapiro–Wilks test, group differences for subcortical, hippocampal subfields, thalamus, and amygdala substructures volumes were examined using multivariate analyses of covariance (MANCOVAs) with one between-subjects grouping factor (groups: TLE-HS, TLE-MRIneg, extra-TLE and HC) with age, gender and estimated total intracranial volume (eTIV) as covariates. The eTIV is a reliable indirect measure of the head size^35^ and is used as a covariate in most large-scale ENIGMA collaborations studies in epilepsy.^26,27,36,37^ All the analyses were followed by Bonferroni *post hoc* correction. To estimate the effect size, independent two-sample *t*-tests were performed between the studied populations, and Cohen’s *d*-value was reported.

Logistic regression was performed to test the relationship between the not flipped volumes of the amygdala structures that resulted significantly different across groups and the clinical diagnosis. Additionally, the accuracy of these models was assessed by areas under the curve (AUCs) with 95% confidence intervals obtained by the receiver operating characteristic curve.

Finally, correlation analyses between the flipped amygdala volumes and clinical variables (age of epilepsy onset, duration of illness, and number of ASMs) were performed for the TLE patients. In the correlations, age, gender and eTIV were included as confounding factors. Independent sample *t*-tests were used to determine whether there were group differences in drug–response and psychiatric comorbidity in relation to the volume of amygdala subnuclei.

All statistical analyses were performed using SPSS software 27 (IBM, Chicago, IL, USA). Statistical significance for all tests was set at *P* < 0.05.

### Standard protocol approval, registration and patient consent

The study was approved by the local Ethical Committee of Area Vasta Emilia Nord (N. 155/14). Patients gave written informed consent for the use of their clinical records in this study. The study was conducted in accordance with the World Medical Association Declaration of Helsinki. The manuscript was prepared according with the STROBE checklist for cross-sectional studies.

### Data availability

The data sets generated during and/or analyzed during the present study are available from the corresponding author on reasonable request.

## Results

### Patients’ population demographic and clinical characteristics

Out of an original pool of 116 TLE patients, 48 were recruited. The remaining 68 patients were excluded for lack of T_1_-3D sequence in the MRI protocol (*n* = 13), progressive neurological diseases (e.g. AD, encephalopathies, *n* = 5), age older than 65 years old (*n* = 5), previous neurosurgery (*n* = 3), structural lesions different from HS (e.g. LEAT ‘Long-term Epilepsy Associated Tumors’, FCD, amygdala signal changes, *n* = 33) and segmentation errors after the FreeSurfer post-processing process (*n* = 9) (Supplementary Fig. 1). Among the TLE group, 24 patients were classified as TLE-HS and 24 as TLE-MRIneg. Demographic and clinical characteristics are summarized in Table 1. The extra-TLE group was constituted by 20 patients, 14 (70%) with frontal lobe epilepsy and 6 (30%) with parietal lobe epilepsy. Twelve of 20 extra-TLE patients (60%) had cryptogenic epilepsy, 6/20 (30%) a frontal FCD, and 2/20 (10%) a FCD in the parietal lobe. Interictal FDG-PET was available in 15 TLE and 7 extra-TLE patients and the revealed hypometabolism confirmed the electro-clinical hypotheses in all cases. Out of all patients’ cohort, seven patients underwent epilepsy surgery: five TLE-HS and two extra-TLE. Histology confirmed the HS in all TLE-HS, and two FCD Type Ia were reported in extra-TLE. In two TLE-HS postsurgical specimens, an amygdala gliosis was documented by the pathologist. Mean follow-up after surgery was 34 months, and all patients are in Engel Class Ia.^38^ No statistical differences were observed between groups in age, gender distribution and eTIV. Across epilepsy groups, there were no statistical differences in the side of the epileptic focus, age at epilepsy onset and epilepsy’s duration. Despite the greater number of drug-resistant patients in the TLE-HS group, the drug–response status did not show a significant difference between the epilepsy groups. There was a statistically significant difference in the number of antiseizure meds between groups: extra-TLE were on polytherapy more frequently compared with TLE-MRIneg (*p*_Bonferroni_ = 0.011). Psychiatric comorbidity was documented in 11 patients mainly represented by TLE: the reported symptoms in all patients were compatible with a mixed anxiety-depressive disorder (MADD).^39^

**Table 1:** Demographic and clinical characteristics of the studied populations

	TLE-MRIneg	TLE-HS (*N* = 24)	Extra-TLE (*N* = 20)	HC (*N* = 30)	*P*-value	Pairwise comparison^a^
Gender, M/F	8/16	9/15	11/9	11/19	0.498^F^	
Age, years	36.54 (13.97)	40.46 (12.13)	34.75 (13.63)	35.27 (5.82)	0.366^K-W^	
Age of onset, yeras	29.21 (14.63)	25.25 (14.54)	21.80 (15.42)	–	0.117^K-W^	
Epilepsy duration, years	7.50 (8.27)	15.29 (12.10)	12.50 (10.89)	–	0.725^K-W^	
Side, L/R	14/10	15/9	10/10	–	0.748^F^	
ASMs-respondents, Yes/no	14/10	6/18	7/13	–	0.054^F^	
No. of ASMs	1.92 (0.88)	2.38 (0.71)	2.55 (0.76)	–	0.031^K-W^*	Extra-TLE > TLE-MRIneg (*P* = 0.011)
Psychiatric comorbidity, Yes/no	3/21	5/19	3/17	–	0.780^F^	
eTIV, mm^3^	1 422 561 (144 470)	1 446 261 (180 209)	1 521 645 (173 721)	1 457 345 (213 170)	0.334^A^	

Data are presented in means, and standard deviations (SDs) are presented in the parentheses. ^F^Fisher’s exact test, ^K-W^Kruskal–Wallis test, ^A^one-way ANOVA. ^a^P-value of pairwise comparisons between groups using Bonferroni method. *P < 0.05.

### Subcortical structures

**Figure 2:**
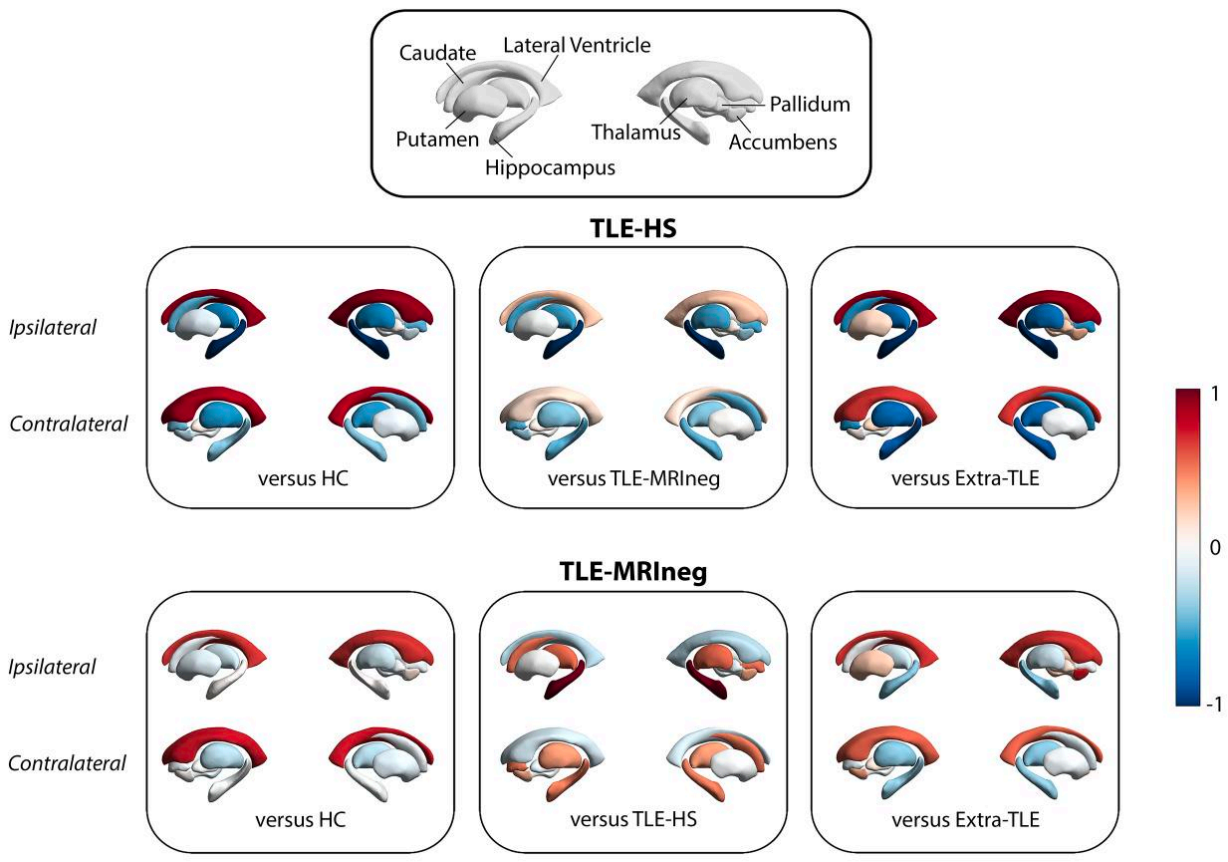
**Graphic representations of subcortical volumes comparison between patients and HC and within patients' populations.** The comparison between TLE-HS and the other groups, and TLE-MRIneg and the other groups are represented with Cohen’s d effect size value starting from absolute z-score volumes. Values close to -1 reflect a decrease of subcortical structure’s volumes while values close to 1 an increase. The top box presents the legend of subcortical structures examined. Present images were created by using the ENIGMA-Toolbox by Larivière *et al*.^40^

The MANCOVA analyses highlighted a decreased volume in the hippocampus ipsilateral to the epileptic focus in TLE-HS compared with all the other groups [TLE-MRIneg, extra-TLE, and HC: *F*(3,91) = 12.498, *P* < 0.000] and an increased lateral ventricular volume bilaterally [*F*(3,91) = 5.561, *P* = 0.002 for the left ventricle; *F*(3,91) = 4.838*, P* = 0.004 for the right ventricle]. An independent sample *t*-test between TLE-HS and HC showed also a significant atrophy of the bilateral thalamus in TLE-HS group compared with HC [left thalamus: *t*(52) = −2.381, *P* = 0.021, *d* = −0.652; right thalamus: *t*(52) = −2.189, *P* = 0.033, *d* = −0.599], whereas no significant differences were observed for basal ganglia and nucleus accumbens. There were no significant differences in subcortical structures between TLE-MRIneg and HC, between extra-TLE and HC and between TLE-MRIneg and extra-TLE groups (Fig. 2).

As far as the hippocampus subfield’s parcellation, the MANCOVA analysis did not find any differences in the volumes of hippocampal structures between TLE-MRIneg, extra-TLE and HC populations. As expected, TLE-HS patients showed overall atrophy of hippocampal subfields ipsilateral to HS compared with all other patients and HC. Few ipsilateral subfields appeared unimpaired by HS: the hippocampal fissure, parasubiculum, fimbria and HATA. The CA4 and dentate gyrus head appeared atrophic even contralaterally. All the results are summarized in Supplementary Table 3. Regarding the thalamus segmentation, the MANCOVA analysis demonstrated isolated atrophy of the ipsilateral mediodorsal magnocellular nucleus in TLE-HS patients compared with all populations (versus HC: *p*_Bonferroni_ = 0.024, versus TLE-MRIneg: *p*_Bonferroni_ = 0.025, versus extra-TLE: *p*_Bonferroni_ = 0.038), and of the ipsilateral anterior portion of the pulvinar compared with HC (*p*_Bonferroni_ = 0.005) (see Supplementary Table 4). No differences were observed between TLE-MRIneg, extra-TLE, and HC (see Supplementary Table 4). An independent sample *t*-test between TLE-HS and HC groups showed a generalized atrophy of whole ipsilateral thalamus [*t*(52) = −2.181, *P* = 0.034, *d* = −0.597] and, particularly, of nuclei belonging to the anterior [anteroventral: *t*(52) = −2.373, *P* = 0.021, *d* = −0.650], intralaminar [central medial: *t*(52) = −2.622, *P* = 0.011, *d* = −0.718; and paracentral: *t*(52) = −2.270, *P* = 0.027, *d* = −0.622], medial group [medial ventral: *t*(52) = −2.308, *P* = 0.025, *d* = -0.631, mediodorsal medial magnocellular: *t*(52) = −3.449, *P* = 0.001, *d* = −0.945, and mediodorsal lateral parvocellular: *t*(52) = −2.697, *P* = 0.009, *d* = −0.739], the whole pulvinar [*t*(52) = −2.341, *P* = 0.023, *d* = −0.641], particularly the anterior [*t*(52) = −3.388, *P* = 0.001, *d* = −0.928] and medial [*t*(52) = −2.471, *P* = 0.017, *d* = −0.677] portions. The same nuclei of the intralaminar and medial group as well as the anterior and medial pulvinar nuclei were atrophic also contralaterally to the HS (Supplementary Table 5).

**Figure 3:**
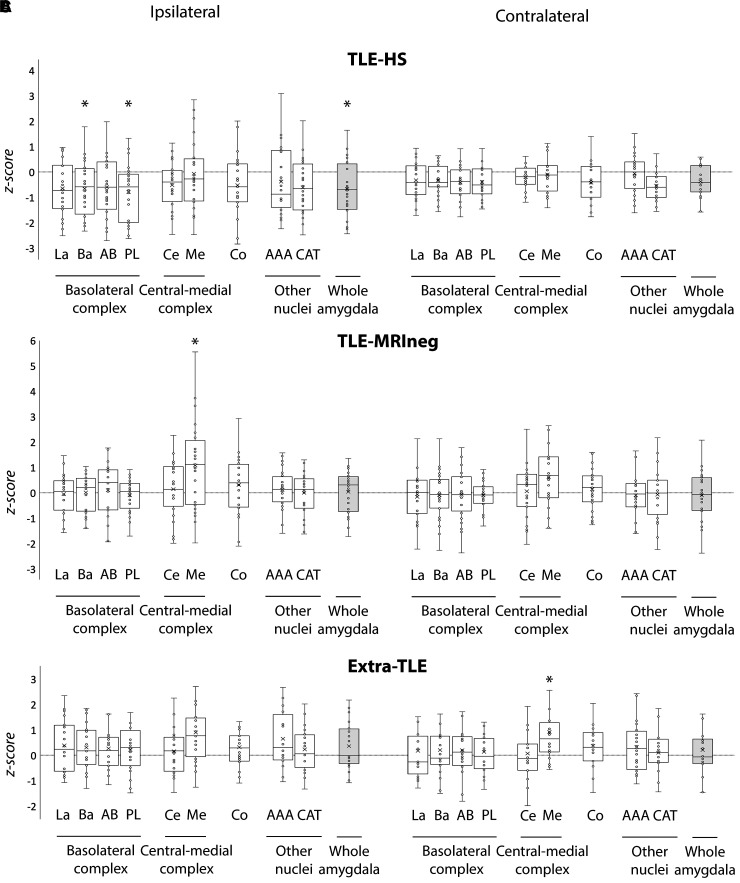
**Amygdalar subnuclei comparisons between patients’ groups and HC.** Box-and-whisker plots of volumes of amygdalar structures ipsilateral and contralateral to the epileptic focus in patients with TLE-HS (**A**), TLE-MRIneg (**B**) and extra-TLE (**C**) standardized relative to HC. The central horizontal line of the boxes marks the median of the sample, the upper and lower edges of the box (the hinges) mark the 25th and 75th percentiles (the central 50% of the values fall within the box). The open circles represent individual patients. The dashed line on value 0 designates the mean volume of HC. The ‘x’ in the middle of each box marks the mean volume for every nucleus. The ‘*’ on the box and/or on the complexes name indicates the significant results of the MANCOVA analysis (*P* < 0.05) of the volume differences between each patients’ group and HC. La, lateral nucleus; Ba, basal nucleus; AB, accessory basal nucleus; PL, paralaminar nucleus; Ce, central nucleus; Me, medial nucleus; Co, cortical nucleus; AAA, anterior amygdaloid area; CAT, corticoamygdaloid transition area.

**Figure 4:**
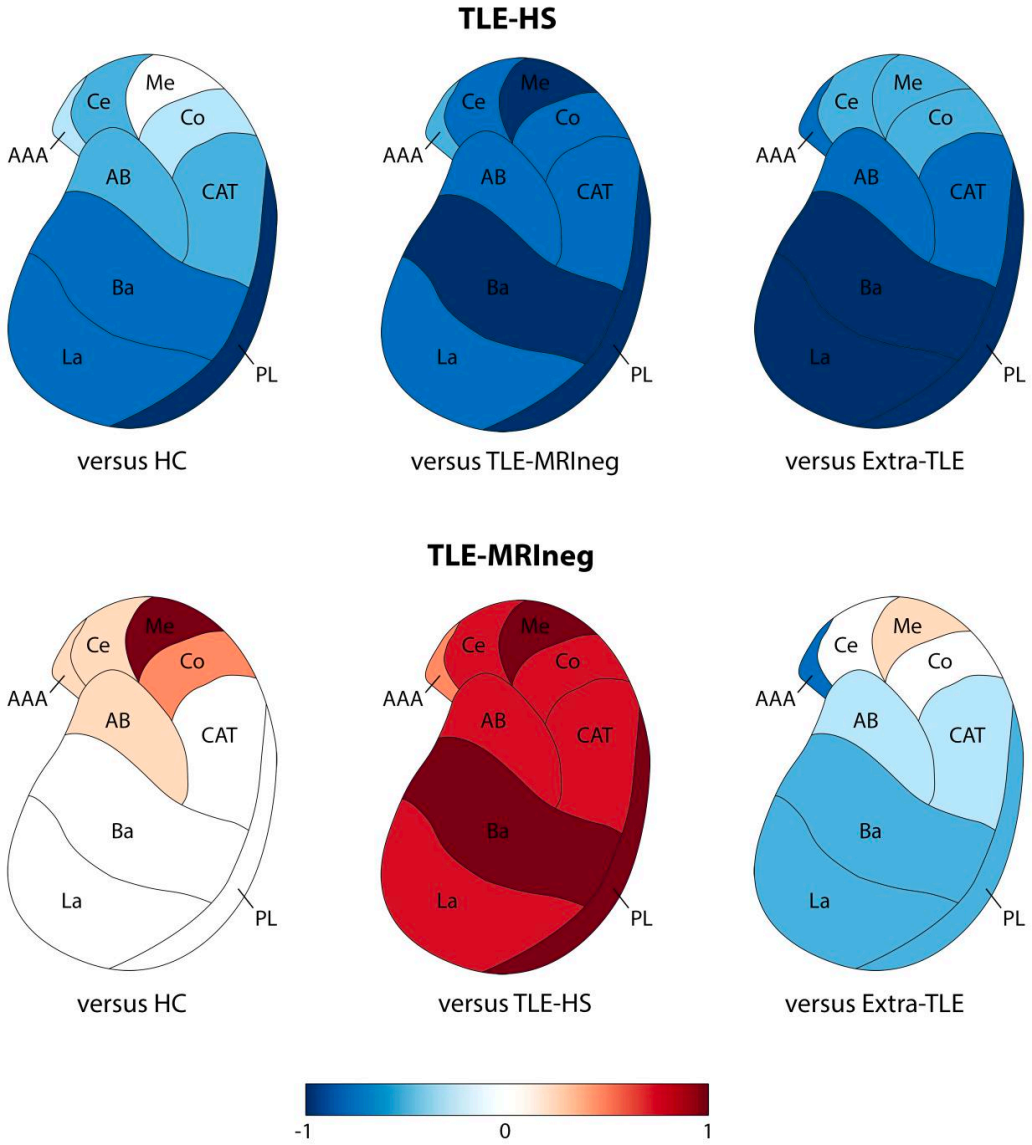
**Graphic representations of amygdalar subnuclei volumes’ comparisons between patients and HC and within patients’ populations.** Only the amygdala ipsilateral to the epileptic focus is presented. The comparison between TLE-HS and the other groups, and TLE-MRIneg and the other groups are represented with Cohen’s d effect size value. Values close to -1 reflect a decrease of volumes in the amygdala subnuclei, while values close to 1 an increase. La, lateral nucleus; Ba, basal nucleus; AB, accessory basal nucleus; PL, paralaminar nucleus; Ce, central nucleus; Me, medial nucleus; Co, cortical nucleus; AAA, anterior amygdaloid area; CAT, corticoamygdaloid transition area. See text for details.

Regarding the amygdala subnuclei and complexes, results are summarized in Table 2 and in Figs 3 and 4. There were no differences in the amygdala volumes between left and right hemispheres in HC (Supplementary Table 6).

**Table 2:** Morphometric comparison of amygdala substructures between patients’ groups and HC

		TLE-MRIneg	TLE-HS	Extra-TLE	HC	*F*	*P*-value	Pairwise comparison^a^
Ipsilateral	Whole amygdala	1821.383 (202.543)*	1657.693 (264.616)*	1902.373 (272.393)*	1799.279 (209.579)*	4.639	0.005**	TLE-HS < HC (*P* = 0.040) TLE-HS < TLE-MRIneg (*P* = 0.010) TLE-HS < exTLE (*P* = 0.018)
	Lateral nucleus	670.808 (68.034)*	619.674 (93.304)*	711.153 (106.522)*	673.051 (77.678)	4.064	0.009**	TLE-HS < TLE-MRIneg (*P* = 0.44) TLE-HS < exTLE (*P* = 0.016)
	Basal nucleus	461.260 (49.973)*	418.723 (70.350)*	481.192 (68.681)*	456.974 (58.518)*	4.320	0.007**	TLE-HS < HC (*P* = 0.042) TLE-HS < TLE-MRIneg (*P* = 0.015) TLE-HS < exTLE (*P* = 0.027)
	AB nucleus	283.686 (41.460)*	256.723 (43.831)*	290.711 (44.443)	276.833 (34.705)	3.833	0.012*	TLE-HS < TLE-MRIneg (*P* = 0.012)
	Paralaminar nucleus	50.994 (4.904)*	45.889 (8.131)*	53.368 (7.486)*	51.643 (6.789)	4.728	0.004**	TLE-HS < HC (*P* = 0.011) TLE-HS < TLE-MRIneg (*P* = 0.016) TLE-HS < exTLE (*P* = 0.024)
	Central nucleus	49.967 (10.638)*	43.936 (8.812)*	49.536 (9.803)	47.455 (7.999)	3.142	0.029*	TLE-HS < TLE-MRIneg (*P* = 0.023)
	Medial nucleus	26.562 (7.952)*	21.313 (6.552)*	26.077 (6.758)	20.747 (4.284)*	5.093	0.003**	TLE-HS < TLE-MRIneg (*P* = 0.009) HC < TLE-MRIneg (*P* = 0.012)
	Cortical nucleus	28.540 (5.278)*	24.819 (5.309)*	28.716 (4.767)	26.837 (4.074)	3.618	0.016*	TLE-HS < TLE-MRIneg (*P* = 0.010)
	AAA	57.656 (7.266)	53.203 (11.486)	62.079 (10.311)	55.937 (7.980)	2.524	0.063	
	CAT	191.825 (23.878)*	174 (32.102)*	198.751 (28.111)	189.961 (27.599)*	3.290	0.024*	TLE-HS < TLE-MRIneg (*P* = 0.037)
	Basolateral complex	366.687 (39.304)*	335.252 (52.614)*	384.106 (55.194)*	364.625 (41.753)*	4.559	0.005**	TLE-HS < HC (*P* = 0.033) TLE-HS < TLE-MRIneg (*P* = 0.013) TLE-HS < exTLE (*P* = 0.018)
	Cortical complex	28.540 (5.278)*	24.819 (5.309)*	28.716 (4.767)	26.837 (4.074)	3.618	0.016*	TLE-HS < TLE-MRIneg (*P* = 0.010)
	Central-medial complex	38.264 (8.744)*	32.625 (7.227)*	37.807 (7.579)	34.101 (5.490)	4.195	0.008**	TLE-HS < TLE-MRIneg (*P* = 0.005)
Contralateral	Whole amygdala	1806.576 (278.917)	1720.756 (164.209)	1878.866 (274.433)	1821.415 (334.747)	1.326	0.271	
	Lateral nucleus	665.074 (97.899)	645.940 (67.732)	692.898 (105.466)	672.753 (120.093)	0.802	0.496	
	Basal nucleus	459.111 (72.231)	438.850 (42.867)	477.929 (72.681)	463.525 (85.591)	1.142	0.336	
	AB nucleus	280.271 (47.547)	262.683 (29.166)	291.781 (44.518)	282.866 (58.423)	1.644	0.185	
	Paralaminar nucleus	51.239 (7.478)	48.679 (5.400)	53.009 (7.730)	51.717 (9.462)	1.648	0.184	
	Central nucleus	50.127 (11.536)	46.674 (6.130)	49.957 (11.332)	49.663 (14.428)	0.637	0.593	
	Medial nucleus	25.694 (7.866)	20.897 (4.043)*	26.702 (6.241)*	22.502 (7.397)*	5.095	0.003**	TLE-HS < exTLE (*P* = 0.017) HC < exTLE (*P* = 0.037)
	Cortical nucleus	28.400 (4.773)	25.096 (4.427)	29.719 (5.141)	27.773 (6.982)	2.962	0.036*	
	AAA	55.857 (9.450)	55.197 (6.812)	59.703 (9.067)	56.643 (10.189)	0.270	0.847	
	CAT	191.219 (33.856)	176.041 (18.329)	196.265 (27.886)	193.981 (34.985)	2.504	0.064	
	Basolateral complex	363.924 (52.249)	349.038 (34.039)	378.904 (56.331)	367.715 (67.395)	1.158	0.330	
	Cortical complex	28.400 (4.773)	25.096 (4.427)	29.719 (5.141)	27.773 (6.982)	2.961	0.036*	
	Central-medial complex	37.911 (8.800)	33.831 (4.426)	38.330 (8.160)	36.083 (10.405)	1.575	0.201	

Data (in mm^3^) are presented in means; standard deviations (SDs) are presented in the parentheses. Age, gender and eTIV as covariates. F, MANCOVA’s *F*-test value. ^a^*P*-value of pairwise comparisons between groups using *post hoc* Bonferroni correction (*P* < 0.05). *P < 0.05, **P < 0.01. AAA, anterior amygdaloid area; CAT, corticoamygdaloid transition area.

An overall atrophy of the whole amygdala, ipsilateral to the epileptic focus, was observed in the TLE-HS versus HC (*p*_Bonferroni_ = 0.040, *d* = −0.613), TLE-MRIneg (*p*_Bonferroni_ = 0.010, *d* = −0.708) and extra-TLE (*p*_Bonferroni_ = 0.018, *d* = −0.958). The atrophy involved particularly the BLA (TLE-HS versus HC, *p*_Bonferroni_ = 0.033, *d* = −0.629; TLE-HS versus TLE-MRIneg, *p*_Bonferroni_ = 0.013, *d* = −0.693; TLE-HS versus extra-TLE, *p*_Bonferroni_ = 0.018, *d* = −0.953) and all its constituent nuclei especially the basal nucleus (TLE-HS versus HC, *p*_Bonferroni_ = 0.042, *d* = −0.620; TLE-HS versus TLE-MRIneg, *p*_Bonferroni_ = 0.015, *d* = −0.723; TLE-HS versus extra-TLE, *p*_Bonferroni_ = 0.027, *d* = −0.942) and the PL (TLE-HS versus HC, *p*_Bonferroni_ = 0.011, *d* = −0.739; TLE-HS versus TLE-MRIneg, *p*_Bonferroni_ = 0.016, *d* = −0.762; TLE-HS versus extra-TLE, *p*_Bonferroni_ = 0.018, *d* = −0.968). The CC and its subnuclei were atrophic in the TLE-HS population versus TLE-MRIneg (*p*_Bonferroni_ = 0.010, *d* = −0.698) ipsilateral to the epileptic focus.

TLE-MRIneg demonstrated a significant increased volume of the medial nucleus (Me) ipsilateral to the epilepsy focus versus HC (*p*_Bonferroni_ = 0.012, *d* = 0.792) and versus TLE-HS (*p*_Bonferroni_ = 0.009, *d* = 0.733). Finally, extra-TLE showed a statistically significant increase of the Me volume contralateral to the epilepsy focus compared with HC (*p*_Bonferroni_ = 0.037, *d* = 0.839) and TLE-HS (*p*_Bonferroni_ = 0.017, *d* = 1.014). Although not significant, it must be noted the whole amygdala volume of TLE-MRIneg and extra-TLE is greater than HC, especially the one ipsilateral to the epileptic focus.

Given the results of MANCOVAs analyses, we explored the behaviour of the BLA and the Me in all the patients’ groups at individual level to isolate the patients in whom the abnormalities were observed. We thus considered abnormal values that were  ± 2 SD from the mean of normal controls. The BLA, ipsilateral to the epileptic focus, was reduced in its volume in 5 of 24 TLE-HS (21%), and no TLE-HS patients showed BLA enlargement. In TLE-MRIneg group, 6 of 24 (25%) patients presented a larger Me ipsilateral to the epileptic focus, while the same nucleus resulted enlarged in three extra-TLE patients (15%), contralateral to the epileptic onset. The Me was never reduced in its volume in any TLE-MRIneg and extra-TLE patients.

Information regarding the subcortical grey matter volume, total grey matter volume and total white matter volume for all groups are summarized in Supplementary Table 7.

### Logistic regression analysis

A multinomial logistic regression analysis showed that the BLA differentiated TLE-HS versus HC (*β* = 0.780, *SE* = 0.297, *P* = 0.009). Sensitivity and specificity of this model were 77 and 71%, respectively (AUC = 0.704), the positive predictive value (PPV) was 77% and the negative predictive value (NPV) was 71%. In TLE-MRIneg, the logistic regression analysis showed the Me of the amygdala was able to discriminate this group from HC (*β* = 0.620, *SE* = 0.221, *P* = 0.005) with a sensitivity and a specificity of 53 and 93% respectively (AUC = 0.714, PPV = 87%, NPV = 72%). In both situations, the AUC measure suggests an acceptable, although not excellent, ability of the BLA and Me volumes to discriminate between patients (TLE-HS and TLE-MRIneg, respectively) and controls. By counterpart, the volumes of both amygdala’s subregions were not able to discriminate between TLE-HS and TLE-MRIneg [Me (*β* = 0.530, *SE* = 0.276, *P* = 0.054), BLA (*β* = 0.133, *SE* = 0.380, *P* = 0.726)].

### Correlation analyses

Age of epilepsy onset, epilepsy duration, number of ASMs, and drug-resistance were not correlated with the amygdala morphometric measures in both TLE-HS and TLE-MRIneg. Although limited by the small number of patients, we tested any significant relation between the presence of psychiatric comorbidity and amygdala volume measures. A significantly increased volume of the ipsilateral lateral nucleus [*t*(22) = 2.117, *P* = 0.046, *d* = 1.307] was observed in TLE-MRIneg patients with psychiatric comorbidity with respect to those without psychiatric disorders. No relations were observed in the entire TLE population between the amygdala subnuclei’s volume and the presence of psychiatric comorbidity.

## Discussion

To the best of our knowledge, this cross-sectional study is the first which utilizes automated neuroanatomical quantification to evaluate in vivo amygdala subnuclei volumetric differences in epilepsy patients. The main findings of the present study are (i) a significant atrophy of the whole amygdala, particularly the BLA in TLE-HS compared with HC and other epilepsy populations and (ii) a significant increased volume the Me (which is part of the CMC) but not the whole amygdala in TLE-MRIneg group compared with HC. In both scenarios the involved amygdala’s structure is ipsilateral to the epileptic focus. Additionally, we observed that the BLA and the Me of amygdala volumes can be differentiated TLE-HS and TLE-MRIneg, respectively, versus HC, with a good performance. Overall, our findings, while confirming the involvement of the amygdala in TLE particularly in patients with mesial TLE with HS, expand previous knowledge as they suggest specific amygdala subnuclei as possible morphological biomarkers of TLE. Indeed, amygdala pathology is of great relevance in view of the importance of this region in the production of a full spectrum of experiential symptoms typical of temporal lobe seizures,^41^ the sensitivity of the amygdala to kindling protocols in animal studies,^42^ and its role in emotional/behavioural alteration in TLE.^43-45^

The involvement of the amygdala in mesial TLE is well recognized especially in association with HS. Histological reports from TLE patients with HS demonstrated in a large proportion the presence of amygdaloid damage represented mainly by neuronal loss and gliosis most often ipsilateral to the HS.^46^ Previous volumetric studies have largely documented an amygdala atrophy in TLE-HS patients on the same side of the sclerotic hippocampus,^47-49^ leading to hypothesize the smallest amygdala as a characteristic report of TLE due to HS.^48^ Our analyses support these observations as a substantial decrease in volume of all the amygdala structures was observed in the TLE-HS population (Figs 3 and 4) compared with HC. Additionally, the present morphometric data show that the volumes’ reductions were ipsilateral to the epileptic focus for all the amygdala nuclei thus sustaining that the volumetric measurements of mesial temporal regions including the amygdala might be useful to the lateralization of the site of seizure onset in TLE.^48^ In our population of TLE-HS patients, the amygdala’s volumes were significantly different not only from HC but even when compared with TLE-MRIneg patients and, for some nuclei belonging to the BLA (see Figs 3 and 4), to extra-TLE subjects. The directionality of this difference is always versus an atrophy in TLE-HS patients. In line with our results, an increase in amygdala’s volume has almost never been documented ipsilaterally to the TLE-HS^7^ while a recent study provided evidence of an AE contralateral to the HS in a proportion of patients with mesial TLE.^3^ As far as the other subcortical structures, in line to what expected, hippocampal subfields were almost all atrophic ipsilaterally to the epileptic focus, thus supporting previous morphometric data in TLE-HS using the same methodological approach.^50-52^ Atrophy was particularly pronounced in CA1-CA4 and dentate gyrus regions as already reported.^50,53^ In our TLE-HS population, CA4 and the dentate gyrus appeared reduced in volume even contralaterally to the HS when compared with controls. Neuronal loss in CA1, CA3, CA4, and in the dentate gyrus has been reported to be typically bilateral in mesial temporal sclerosis, although the atrophy is greater on the side of the epileptic focus.^53^ Beyond the hippocampus, we demonstrated a bilateral thalamic atrophy coupled with a bilateral lateral ventricle enlargement. These data are in line with a growing body of literature^26,54-56^ indicating that TLE-HS is an example of network disease in which atrophy extends beyond the mesial temporal regions. Additionally, the thalamic nuclei’s segmentation demonstrated that the volumetric changes are mostly homolateral to the HS, in agreement with post-mortem anatomic-pathology evidence.^57,58^ Intriguingly, our analysis shown that in HS patients, the nuclei belonging to the so-called ‘limbic thalamus’ were mainly involved.^59^ Wrist the pulvinar is not formally part of the limbic network, its involvement has been reported in mesial TLE epilepsies by intracerebral electrophysiological recordings,^60-62^ imaging studies,^63^ and the entity of its atrophy linked to the resistance to epilepsy surgery.^55^ Since hippocampus and thalamus in TLE patients present specific morphometric patterns that have been largely documented,^26,52,58,64,65^ the results obtained by these additional analyses on hippocampus’ subfields and thalamus’ subnuclei reinforce the assumption that our TLE sample is representative of the general TLE population, and the amygdala subnuclei analyses are consequently reliable.

TLE MRI-negative patients represent a clinical challenge especially within the presurgical work-up. An increased volume of amygdala (i.e. AE) was found in MRI-negative TLE in several reports and interpreted as possible epileptogenic focus.^6,7,10^ By contrast, in our analysis the whole amygdala’s volumes, while appearing greater in TLE-MRIneg and extra-TLE with respect to controls, did not reach the statistical significance except for the Me of amygdala which resulted hypertrophic in both populations. It must be noted that the mean age at seizure’s onset in our population were lower compared with previous studies.^66^ This is not trivial, as it has been shown by others a relationship between AE and a later epilepsy onset.^12,66^ In addition, AE in older age is more likely to be associated with a faster resolution of the AE at follow-up thus possibly reflecting inflammatory/encephalitis processes or seizures-induced changes.^4^ The individual level analysis confirmed the hypertrophy of the Me in 25% of TLE-MRIneg and in 15% of extra-TLE patients, while this nucleus was never atrophic in both populations. These rates of amygdala volume changes, although limited to single subnuclei/complexes, are in line with previously reported percentage of AE in non-lesional TLE patients.^3,10,11^ Overall, the present analyses support and expand previous observations in patients with non-lesional focal epilepsy,^11^ by confirming that the increased volume of amygdala represents an unspecific finding common across different epilepsy syndromes, probably not limited to MRI-negative cases.

### Different amygdala subnuclei involvement in TLE

Among the various nuclei of amygdala, the lateral and the BLA have been demonstrated to display the greatest histochemical^67^ and pathological alterations^68^ in patients with mesial TLE. The BLA is constituted of the lateral nucleus, the basal nucleus, the AB, and the PL, and it comprises 69% of the total amygdala volume in humans.^69^ This complex of the amygdala (i.e. BLA) receives strong sensory input from multiple cortical and thalamic sources,^70,71^ which terminate primarily in the lateral nucleus, and has reciprocal interactions with the hippocampal formation,^72^ including the entorhinal cortex, perirhinal cortex and parahippocampal cortex. According to a recent study,^73^ the BLA and hippocampus generate a circuit of information, and the connection area of BLA with the hippocampus is typically the CA1 subregion which is one of the most vulnerable fields for gliosis and neuronal loss as observed in the HS.^73,74^ Our results of a pronounced basolateral atrophy in TLE-HS ipsilateral to the epileptic focus support previous described electrophysiological and histochemical studies. The exact mechanism by which the amygdala activity can interplay with the atrophic hippocampus and contribute to seizures generation/maintenance is not completely clear, but evidence suggests an excitation-inhibition unbalance toward a disinhibited state^67,75^ of the amygdala substructures.

In the TLE-MRIneg group the Me was increased in its volume, ipsilateral to the epileptic focus. This nucleus belongs to the CMC and represents the main output of the amygdala to the brainstem and hypothalamus.^72^ Human neuroimaging studies support a role of the CMC in motor behaviour and response preparation and throughout its connections with the hypothalamus and brainstem mediates the visceral and autonomic reactions to fear.^76^ Additionally, recent resting state fMRI studies provided evidence about its involvement also in the emotional processing, social behaviour and executive control process mediated by direct connection with the ventromedial frontal cortex.^77^ Future studies must integrate neuropsychological data and amygdala morphometric analysis to better understand if the involvement of different amygdala nuclear complexes is related to specific cognitive-behavioural impairments in larger cohorts of patients. As an interesting and speculative observation, the Me appeared hypertrophic even in extra-TLE patients, although contralateral to the epilepsy focus. The observation of an amygdala involvement ipsilateral to the epileptic focus in TLE while contralateral to extra-TLE deserve further investigations, on higher number of subjects, and correlations with epilepsy and behavioural patients’ phenotypes.

### Relationship between amygdala subnuclear volumes and clinical variables

Correlation analyses did not disclose any significant relationship between the amygdala’s subnuclei volumes and the clinical variables including age of epilepsy onset, duration of epilepsy, drug–response status in the last 12 months, and ASMs load in TLE-HS and TLE-MRIneg. Similar findings have been observed by others in a larger cohort of patients with AE.^10,11^

A remarkable result of this study was the correlation between the increased volume of the ipsilateral lateral nucleus (which is part of the BLA), in those patients with TLE-MRIneg and MADD symptoms. Despite the caution due to the small number of patients, this finding is of interest being consistent with previous volumetric studies in TLE patients with psychosis and dysthymia symptoms.^78,79^ Patients with psychosis and epilepsy showed indeed an AE compared with TLE without psychiatric symptoms.^79^ The basolateral amygdala integrates inputs from sensory and other limbic structures and has been theorized to function as ‘gate-keeper’ by assessing incoming sensory information and assigning emotional saliency to appropriate stimuli. In addition, alteration of the BLA might affect downstream pathways involved in social cognition and decision-making processes throughout its communication with the prefrontal and orbitofrontal cortex.^80^ Morphometric alterations of BLA have been documented in vivo in different psychiatric conditions.^16,18,81,82^ Particularly, in patients with psychosis, the basolateral amygdala, and particularly the lateral nucleus, resulted affected, confirming the role of this structure in schizophrenia patients, and highlighting an alteration of its volume as a biomarker even in unaffected but high-risk subjects.^17^

An interesting experiment in animals’ models documented that BLA is selectively affected by chronic stress and present microstructural alterations including dendritic hypertrophy and spine enlargement; these changes in turn correlated to the anxiety-like behaviours of the animals.^83^ Recently, an enlargement of the BLA was positively correlated with social and communication impairments in adolescents with autism^84^ and the deep brain stimulation of the BLA has been proposed as a possible treatment for social anxiety.^80^

### Study limitations

We are aware that this study has limitations. Firstly, we recognize the limited sample of patients studied in the TLE and extra-TLE subgroups. However, this limitation is justified by the strict inclusion criteria adopted. We explored indeed the amygdala subnuclei volumes being careful to exclude patients with any sign of amygdala abnormalities on structural MRI, both in terms of altered volume and/or signal. MRI scans, including the oldest ones, were acquired using dedicated epilepsy protocols and inspected by expert neuroradiologists. Compatibly with the limitation of visual inspection, we are thus confident to have collected real ‘negative MRI’ TLE cases. Secondly, the amygdala subnuclei segmentation adopted here has not previously been used in the epilepsy contest which raises concerns about the reliability of the approach adopted. Segmentation of amygdala nuclei is challenging due to small regional volumes and limited availability of a clear ground truth. The amygdala atlas was developed by manually segmenting amygdala in post-mortem samples using high-resolution 7T MRI. Since its introduction,^14^ the algorithm has been validated in different contests including psychiatric disorders^16-18^ and premature born adults.^85^ Recently, Buser *et al*.^86^ explored specifically the spatial and numerical reliability for the segmentation of amygdala and hippocampal nuclei in FreeSurfer. The numerical reliability was mostly high within all the amygdala subnuclei except for a few regions including the AAA and the PL, which demonstrated only moderate spatial reliability. Thirdly, medication could influence the amygdala and subnuclei volumes of TLE and extra-TLE patients. In this study, all the patients were taking at least one ASM and mostly more than one, so we could not rule out drug effects on the results. However, correlation analysis did not disclose any relationship between the amygdala subnuclear volumes and the ASM drug-load. Fourth, this study is a cross-sectional study. Future studies would benefit from longitudinal monitoring to determine whether the amygdala and subnuclei volumes change during the individual’s clinical progression. Different studies indeed reported a decreased volume of enlarged amygdala at follow-up visits in parallel with achieving seizures’ freedom, suggesting that at least in some patients, amygdala hypertrophy can be linked to seizures’ recurrence.^4,87^

## Supplementary Material

fcac225_Supplementary_Data

## Abbreviations

AAA =: anterior amygdaloid area
AB =: accessory basal nucleus
AE =: amygdala enlargement
ASM =: antiseizures medication
AUC =: area under the curve
Ba =: basal nucleus
BLA =: basolateral complex
CAT =: corticoamygdaloid transition area
CC =: cortical complex
Ce =: central nucleus
CMC =: central–medial complex
Co =: cortical nucleus
eTIV =: estimated total intracranial volume
Extra-TLE =: extratemporal lobe epilepsy
FCD =: focal cortical dysplasia
FLAIR =: fluid-attenuated inversion recovery
GC-ML-GD =: granule cell and molecular layer of the dentate gyrus
HATA =: hippocampal-amygdala transition area
HCs =: healthy controls
La =: lateral nucleus
MADD =: mixed anxiety-depressive disorder
Me =: medial nucleus
PL =: paralaminar nucleus
TLE =: temporal lobe epilepsy
TLE-HS =: temporal lobe epilepsy with hippocampal sclerosis
TLE-MRIneg =: temporal lobe epilepsy with negative MRI

## References

[1] Salzman CD, Fusi S. Emotion, cognition, and mental state representation in amygdala and prefrontal cortex. Annu Rev Neurosci. 2010;33:173–202. 20331363 10.1146/annurev.neuro.051508.135256 PMC3108339

[2] Wieser HG. Mesial temporal lobe epilepsy with hippocampal sclerosis. Epilepsia. 2004;45(6):695–714. 15144438 10.1111/j.0013-9580.2004.09004.x

[3] Coan AC, Morita ME, Campos BM, Bergo FPG, Kubota BY, Cendes F. Amygdala enlargement occurs in patients with mesial temporal lobe epilepsy and hippocampal sclerosis with early epilepsy onset. Epilepsy Behav. 2013;29(2):390–394. 24074891 10.1016/j.yebeh.2013.08.022

[4] Na HK, Lee H, Hong S, et al Volume change in amygdala enlargement as a prognostic factor in patients with temporal lobe epilepsy: A longitudinal study. Epilepsia. 2020;61(1):70–80. 31828789 10.1111/epi.16400

[5] Morita-Sherman M, Li M, Joseph B, et al Incorporation of quantitative MRI in a model to predict temporal lobe epilepsy surgery outcome. Brain Commun 2021;3(3):fcab164. 34396113 10.1093/braincomms/fcab164 PMC8361423

[6] Lv RJ, Sun ZR, Cui T, Guan HZ, Ren HT, Shao XQ. Temporal lobe epilepsy with amygdala enlargement: A subtype of temporal lobe epilepsy. BMC Neurol 2014;14(1):194. 25269594 10.1186/s12883-014-0194-z PMC4210593

[7] Mitsueda-Ono T, Ikeda A, Inouchi M, et al Amygdalar enlargement in patients with temporal lobe epilepsy. J Neurol Neurosurg Psychiatry 2011;82(6):652–657. 21047879 10.1136/jnnp.2010.206342

[8] Minami N, Morino M, Uda T, et al Surgery for amygdala enlargement with mesial temporal lobe epilepsy: Pathological findings and seizure outcome. J Neurol Neurosurg Psychiatry 2015;86(8):887–894. 25224675 10.1136/jnnp-2014-308383

[9] Kim DW, Lee SK, Chung CK, Koh YC, Choe G, Lim SD. Clinical features and pathological characteristics of amygdala enlargement in mesial temporal lobe epilepsy. J Clin Neurosci 2012;19(4):509–512. 22321366 10.1016/j.jocn.2011.05.042

[10] Coan AC, Morita ME, de Campos BM, Yasuda CL, Cendes F. Amygdala enlargement in patients with mesial temporal lobe epilepsy without hippocampal sclerosis. Front Neurol 2013;4:166. 24298266 10.3389/fneur.2013.00166 PMC3829468

[11] Reyes A, Thesen T, Kuzniecky R, et al Amygdala enlargement: Temporal lobe epilepsy subtype or nonspecific finding? Epilepsy Res. 2017;132:34–40. 28284051 10.1016/j.eplepsyres.2017.02.019 PMC5945291

[12] Bower SPC. Amygdala volumetry in “imaging-negative” temporal lobe epilepsy. J Neurol Neurosurg Psychiatry. 2003;74(9):1245–1249. 12933928 10.1136/jnnp.74.9.1245 PMC1738652

[13] Janak PH, Tye KM. From circuits to behaviour in the amygdala. Nature. 2015;517(7534):284–292. 25592533 10.1038/nature14188 PMC4565157

[14] Saygin ZM, Kliemann D, Iglesias JE, et al High-resolution magnetic resonance imaging reveals nuclei of the human amygdala: Manual segmentation to automatic atlas. NeuroImage. 2017;155:370–382. 28479476 10.1016/j.neuroimage.2017.04.046 PMC5557007

[15] Sah P, Faber ESL, Lopez De Armentia M, Power J. The amygdaloid complex: Anatomy and physiology. Physiol Rev. 2003;83(3):803–834. 12843409 10.1152/physrev.00002.2003

[16] Asami T, Nakamura R, Takaishi M, et al Smaller volumes in the lateral and basal nuclei of the amygdala in patients with panic disorder. PLoS One. 2018;13(11):e0207163. 30403747 10.1371/journal.pone.0207163 PMC6221356

[17] Armio RL, Laurikainen H, Ilonen T, et al Amygdala subnucleus volumes in psychosis high-risk state and first-episode psychosis. Schizophr Res. 2020;215:284–292. 31744752 10.1016/j.schres.2019.10.014

[18] Cui D, Guo Y, Cao W, et al Correlation between decreased amygdala subnuclei volumes and impaired cognitive functions in pediatric bipolar disorder. Front Psychiatry. 2020;11:612. 32670120 10.3389/fpsyt.2020.00612 PMC7332860

[19] Mariajoseph FP, Muthusamy S, Amukotuwa S, Seneviratne U. Seizure-induced reversible MRI abnormalities in patients with single seizures: A systematic review. Epileptic Disord Int Epilepsy J Videotape 2021;23(4):552–562. 10.1684/epd.2021.1300 34240708

[20] Fisher RS, Cross JH, French JA, et al Operational classification of seizure types by the international league against epilepsy: Position paper of the ILAE commission for classification and terminology. Epilepsia. 2017;58(4):522–530. 28276060 10.1111/epi.13670

[21] Scheffer IE, Berkovic S, Capovilla G, et al ILAE Classification of the epilepsies: Position paper of the ILAE commission for classification and terminology. Epilepsia. 2017;58(4):512–521. 28276062 10.1111/epi.13709 PMC5386840

[22] Kwan P, Arzimanoglou A, Berg AT, et al Definition of drug resistant epilepsy: Consensus proposal by the ad hoc task force of the ILAE commission on therapeutic strategies. Epilepsia. 2010;51(6):1069–1077. 19889013 10.1111/j.1528-1167.2009.02397.x

[23] Duncan JS. Seizure-induced neuronal injury: Human data. Neurology 2002:59(9 Suppl 5):S15–S20. 10.1212/wnl.59.9_suppl_5.s15 12428027

[24] Hibar DP, Westlye LT, Doan NT, et al Cortical abnormalities in bipolar disorder: An MRI analysis of 6503 individuals from the ENIGMA bipolar disorder working group. Mol Psychiatry. 2018;23(4):932–942. 28461699 10.1038/mp.2017.73 PMC5668195

[25] Stein M, Winkler C, Kaiser A, Dierks T. Structural brain changes related to bilingualism: Does immersion make a difference? Front Psychol. 2014;5:1116. 25324816 10.3389/fpsyg.2014.01116 PMC4183087

[26] Whelan CD, Altmann A, Botía JA, et al Structural brain abnormalities in the common epilepsies assessed in a worldwide ENIGMA study. Brain. 2018;141(2):391–408. 29365066 10.1093/brain/awx341 PMC5837616

[27] Park BY, Lariviere S, Rodríguez-Cruces R, et al Topographic divergence of atypical cortical asymmetry and regional atrophy patterns in temporal lobe epilepsy: A worldwide ENIGMA study. Brain. 2021;145:1285–1298. 10.1093/brain/awab417 PMC9128824 35333312

[28] Boedhoe PSW, Schmaal L, Abe Y, et al Cortical abnormalities associated with pediatric and adult obsessive-compulsive disorder: Findings from the ENIGMA obsessive-compulsive disorder working group. Am J Psychiatry. 2018;175(5):453–462. 29377733 10.1176/appi.ajp.2017.17050485 PMC7106947

[29] Schmaal L, Veltman DJ, van Erp TGM, et al Subcortical brain alterations in major depressive disorder: Findings from the ENIGMA major depressive disorder working group. Mol Psychiatry. 2016;21(6):806–812. 26122586 10.1038/mp.2015.69 PMC4879183

[30] Price JL, Russchen FT, Amaral DG. The limbic region. II: The amygdaloid complex. Elsevier Science; 1987.

[31] Iglesias JE, Augustinack JC, Nguyen K, et al A computational atlas of the hippocampal formation using ex vivo, ultra-high resolution MRI: Application to adaptive segmentation of in vivo MRI. NeuroImage. 2015;115:117–137. 25936807 10.1016/j.neuroimage.2015.04.042 PMC4461537

[32] Iglesias JE, Insausti R, Lerma-Usabiaga G, et al A probabilistic atlas of the human thalamic nuclei combining ex vivo MRI and histology. Neuroimage. 2018;183:314–332. 30121337 10.1016/j.neuroimage.2018.08.012 PMC6215335

[33] Johnson WE, Li C, Rabinovic A. Adjusting batch effects in microarray expression data using empirical Bayes methods. Biostatistics. 2007;8(1):118–127. 16632515 10.1093/biostatistics/kxj037

[34] Fortin JP, Cullen N, Sheline YI, et al Harmonization of cortical thickness measurements across scanners and sites. NeuroImage. 2018;167:104–120. 29155184 10.1016/j.neuroimage.2017.11.024 PMC5845848

[35] Hansen TI, Brezova V, Eikenes L, Håberg A, Vangberg TR. How does the accuracy of intracranial volume measurements affect normalized brain volumes? Sample size estimates based on 966, subjects from the HUNT MRI cohort. Am J Neuroradiol. 2015;36(8):1450–1456. 25857759 10.3174/ajnr.A4299 PMC7964698

[36] Larivière S, Rodríguez-Cruces R, Royer J, et al Network-based atrophy modeling in the common epilepsies: A worldwide ENIGMA study. Sci Adv. 2020;6(47):eabc6457. 33208365 10.1126/sciadv.abc6457 PMC7673818

[37] Sisodiya SM, Whelan CD, Hatton SN, et al The ENIGMA-epilepsy working group: Mapping disease from large data sets. Hum Brain Mapp. 2020;43:113–128. 10.1002/hbm.25037 PMC8675408 32468614

[38] Engel J. Surgical treatment of epilepsies. 2nd edn. Raven Press; 1993.

[39] Kara S, Yazici KM, Güleç C, Unsal I. Mixed anxiety-depressive disorder and major depressive disorder: Comparison of the severity of illness and biological variables. Psychiatry Res. 2000;94(1):59–66. 10788678 10.1016/s0165-1781(00)00131-1

[40] Larivière S, Paquola C, Park B-Y, et al The ENIGMA toolbox: Multiscale neural contextualization of multisite neuroimaging datasets. Nat Methods. 2021;18(7):698–700. 34194050 10.1038/s41592-021-01186-4 PMC8983056

[41] Gloor P, Olivier A, Quesney LF, Andermann F, Horowitz S. The role of the limbic system in experiential phenomena of temporal lobe epilepsy. Ann Neurol. 1982;12(2):129–144. 7125603 10.1002/ana.410120203

[42] Goddard GV. Analysis of avoidance conditioning following cholinergic stimulation of amygdala in rats. J Comp Physiol Psychol. 1969;68(2):1–18. 10.1037/h0027504 5784697

[43] Bonora A, Benuzzi F, Monti G, et al Recognition of emotions from faces and voices in medial temporal lobe epilepsy. Epilepsy Behav. 2011;20:648–654. 21459049 10.1016/j.yebeh.2011.01.027

[44] Meletti S, Benuzzi F, Rubboli G, et al Impaired facial emotion recognition in early-onset right mesial temporal lobe epilepsy. Neurology. 2003;60(3):426–431. 12578923 10.1212/wnl.60.3.426

[45] Monti G, Meletti S. Emotion recognition in temporal lobe epilepsy: A systematic review. Neurosci Biobehav Rev. 2015;55:280–293. 25999121 10.1016/j.neubiorev.2015.05.009

[46] Hudson LP, Munoz DG, Miller L, McLachlan RS, Girvin JP, Blume WT. Amygdaloid sclerosis in temporal lobe epilepsy. Ann Neurol. 1993;33(6):622–631. 8498843 10.1002/ana.410330611

[47] Bernasconi N. Mesial temporal damage in temporal lobe epilepsy: A volumetric MRI study of the hippocampus, amygdala and parahippocampal region. Brain. 2003;126(2):462–469. 12538412 10.1093/brain/awg034

[48] Cendes F, Andermann F, Gloor P, et al MRI Volumetric measurement of amygdala and hippocampus in temporal lobe epilepsy. Neurology. 1993;43(4):719–725. 8469329 10.1212/wnl.43.4.719

[49] Graebenitz S, Kedo O, Speckmann EJ, et al Interictal-like network activity and receptor expression in the epileptic human lateral amygdala. Brain J Neurol. 2011;134(Pt 10):2929–2947. 10.1093/brain/awr202 21893592

[50] Costa BS, Santos MCV, Rosa DV, Schutze M, Miranda DM, Romano-Silva MA. Automated evaluation of hippocampal subfields volumes in mesial temporal lobe epilepsy and its relationship to the surgical outcome. Epilepsy Res. 2019;154:152–156. 31153103 10.1016/j.eplepsyres.2019.05.011

[51] Ono SE, Mader-Joaquim MJ, de Carvalho Neto A, de Paola L, dos Santos GR, Silvado CES. Relationship between hippocampal subfields and verbal and visual memory function in mesial temporal lobe epilepsy patients. Epilepsy Res. 2021;175:106700. 34175793 10.1016/j.eplepsyres.2021.106700

[52] Riederer F, Seiger R, Lanzenberger R, et al Automated volumetry of hippocampal subfields in temporal lobe epilepsy. Epilepsy Res. 2021;175:106692. 34175792 10.1016/j.eplepsyres.2021.106692

[53] Sadler RM. The syndrome of mesial temporal lobe epilepsy with hippocampal sclerosis: Clinical features and differential diagnosis. Adv Neurol. 2006;97:27–37. 16383112

[54] Bonilha L, Montenegro MA, Cendes F, Li LM. The role of neuroimaging in the investigation of patients with single seizures, febrile seizures, or refractory partial seizures. Med Sci Monit. 2004;10(3):RA40–RA46. 14976443

[55] Keller SS, O’Muircheartaigh J, Traynor C, Towgood K, Barker GJ, Richardson MP. Thalamotemporal impairment in temporal lobe epilepsy: A combined MRI analysis of structure, integrity, and connectivity. Epilepsia. 2014;55(2):306–315. 24447099 10.1111/epi.12520 PMC4074767

[56] de Campos BM, Coan AC, Lin Yasuda C, Casseb RF, Cendes F. Large-scale brain networks are distinctly affected in right and left mesial temporal lobe epilepsy. Hum Brain Mapp. 2016;37(9):3137–3152. 27133613 10.1002/hbm.23231 PMC5074272

[57] Sinjab B, Martinian L, Sisodiya SM, Thom M. Regional thalamic neuropathology in patients with hippocampal sclerosis and epilepsy: A postmortem study. Epilepsia 2013;54(12):2125–2133. 24138281 10.1111/epi.12403 PMC3995016

[58] Lee HJ, Seo SA, Park KM. Quantification of thalamic nuclei in patients diagnosed with temporal lobe epilepsy and hippocampal sclerosis. Neuroradiology. 2020;62(2):185–195. 31673749 10.1007/s00234-019-02299-6

[59] Vertes RP, Linley SB, Hoover WB. Limbic circuitry of the midline thalamus. Neurosci Biobehav Rev. 2015;54:89–107. 25616182 10.1016/j.neubiorev.2015.01.014 PMC4976455

[60] Guye M, Régis J, Tamura M, et al The role of corticothalamic coupling in human temporal lobe epilepsy. Brain. 2006;129(7):1917–1928. 16760199 10.1093/brain/awl151

[61] Rosenberg DS, Mauguière F, Demarquay G, et al Involvement of medial pulvinar thalamic nucleus in human temporal lobe seizures. Epilepsia. 2006;47(1):98–107. 16417537 10.1111/j.1528-1167.2006.00375.x

[62] Rosenberg DS, Mauguière F, Catenoix H, Faillenot I, Magnin M. Reciprocal thalamocortical connectivity of the medial pulvinar: A depth stimulation and evoked potential study in human brain. Cereb Cortex. 2009;19(6):1462–1473. 18936272 10.1093/cercor/bhn185

[63] Barron DS, Eickhoff SB, Clos M, Fox PT. Human pulvinar functional organization and connectivity. Hum Brain Mapp. 2015;36(7):2417–2431. 25821061 10.1002/hbm.22781 PMC4782796

[64] Park KM, Kim TH, Mun CW, et al Reduction of ipsilateral thalamic volume in temporal lobe epilepsy with hippocampal sclerosis. J Clin Neurosci. 2018;55:76–81. 29958756 10.1016/j.jocn.2018.06.025

[65] Wu D, Chang F, Peng D, Xie S, Li X, Zheng W. The morphological characteristics of hippocampus and thalamus in mesial temporal lobe epilepsy. BMC Neurol. 2020;20:235. 32513122 10.1186/s12883-020-01817-x PMC7282186

[66] Beh SMJ, Cook MJ, D’Souza WJ. Isolated amygdala enlargement in temporal lobe epilepsy: A systematic review. Epilepsy Behav. 2016;60:33–41. 27176882 10.1016/j.yebeh.2016.04.015

[67] Yilmazer-Hanke DM, Wolf HK, Schramm J, Elger CE, Wiestler OD, Blümcke I. Subregional pathology of the amygdala complex and entorhinal region in surgical specimens from patients with pharmacoresistant temporal lobe epilepsy. J Neuropathol Exp Neurol. 2000;59(10):907–920. 11079781 10.1093/jnen/59.10.907

[68] Aliashkevich AF, Yilmazer-Hanke D, Van Roost D, Mundhenk B, Schramm J, Blümcke I. Cellular pathology of amygdala neurons in human temporal lobe epilepsy. Acta Neuropathol. 2003;106(2):99–106. 12684832 10.1007/s00401-003-0707-0

[69] LeDoux J. The amygdala. Curr Biol. 2007;17(20):R868–R874. 17956742 10.1016/j.cub.2007.08.005

[70] Aggleton JP, Burton MJ, Passingham RE. Cortical and subcortical afferents to the amygdala of the rhesus monkey (*Macaca mulatta*). Brain Res. 1980;190(2):347–368. 6768425 10.1016/0006-8993(80)90279-6

[71] McDonald AJ. Cortical pathways to the mammalian amygdala. Prog Neurobiol. 1998;55(3):257–332. 9643556 10.1016/s0301-0082(98)00003-3

[72] Benarroch EE. The amygdala: Functional organization and involvement in neurologic disorders. Neurology. 2015;84(3):313–324. 25527268 10.1212/WNL.0000000000001171

[73] Yang Y, Wang JZ. From structure to behavior in basolateral amygdala-hippocampus circuits. Front Neural Circuits. 2017;11:86. 29163066 10.3389/fncir.2017.00086 PMC5671506

[74] Dutra JR, Cortés EP, Vonsattel JPG. Update on hippocampal sclerosis. Curr Neurol Neurosci Rep. 2015;15(10):67. 26299276 10.1007/s11910-015-0592-7

[75] Kullmann DM. What’s wrong with the amygdala in temporal lobe epilepsy? Brain. 2011;134(10):2800–2801. 21921018 10.1093/brain/awr246 PMC3187545

[76] Petrulis A. Chapter 2 - structure and function of the medial amygdala. In: Handbook of amygdala structure and function, Vol. 29. 2020:39–61.

[77] Kerestes R, Chase HW, Phillips ML, Ladouceur CD, Eickhoff SB. Multimodal evaluation of the amygdala’s functional connectivity. NeuroImage. 2017;148:219–229. 28089676 10.1016/j.neuroimage.2016.12.023 PMC5416470

[78] van Elst LT, Woermann FG, Lemieux L, Trimble MR. Amygdala enlargement in dysthymia–a volumetric study of patients with temporal lobe epilepsy. Biol Psychiatry. 1999;46(12):1614–1623. 10624542 10.1016/s0006-3223(99)00212-7

[79] Van Elst LT, Baeumer D, Lemieux L, et al Amygdala pathology in psychosis of epilepsy: A magnetic resonance imaging study in patients with temporal lobe epilepsy. Brain J Neurol. 2002;125(Pt 1):140–149. 10.1093/brain/awf008 11834599

[80] Sinha S, McGovern RA, Sheth SA. Deep brain stimulation for severe autism: From pathophysiology to procedure. Neurosurg Focus. 2015;38(6):E3. 10.3171/2015.3.FOCUS1548 26030703

[81] Zhang L, Hu X, Lu L, et al Anatomic alterations across amygdala subnuclei in medication-free patients with obsessive–compulsive disorder. J Psychiatry Neurosci. 2020;45(5):334–343. 32293840 10.1503/jpn.190114 PMC7850150

[82] Aghamohammadi-Sereshki A, Coupland NJ, Silverstone PH, et al Effects of childhood adversity on the volumes of the amygdala subnuclei and hippocampal subfields in individuals with major depressive disorder. J Psychiatry Neurosci. 2021;46(1):E186–E195. 33497169 10.1503/jpn.200034 PMC7955852

[83] Zhang JY, Liu TH, He Y, et al Chronic stress remodels synapses in an amygdala circuit-specific manner. Biol Psychiatry. 2019;85(3):189–201. 30060908 10.1016/j.biopsych.2018.06.019 PMC6747699

[84] Seguin D, Pac S, Wang J, Nicolson R, Martinez-Trujillo J, Duerden EG. Amygdala subnuclei development in adolescents with autism spectrum disorder: Association with social communication and repetitive behaviors. Brain Behav. 2021;11(8):e2299. 34333868 10.1002/brb3.2299 PMC8413788

[85] Schmitz-Koep B, Zimmermann J, Menegaux A, et al Within amygdala: Basolateral parts are selectively impaired in premature-born adults. NeuroImage Clin. 2021;31:102780. 34391140 10.1016/j.nicl.2021.102780 PMC8374486

[86] Kahhale I, Buser NJ, Madan CR, Hanson JL. Quantifying numerical and spatial reliability of amygdala and hippocampal subdivisions in FreeSurfer. bioRxiv 2020.06.12.149203. 2020. 10.1186/s40708-023-00189-5 PMC10082143 37029203

[87] Peedicail JS, Sandy S, Singh S, et al Long term sequelae of amygdala enlargement in temporal lobe epilepsy. Seizure. 2020;74:33–40. 31812090 10.1016/j.seizure.2019.11.015

