# Supplementary material for: Amygdala subnuclear volumes in temporal lobe epilepsy with hippocampal sclerosis and in non-lesional patients
Source: Brain Commun. 2022 Sep 6;4(5):fcac225. doi: 10.1093/braincomms/fcac225 (PMC9536297; doi:10.1093/braincomms/fcac225)
Supplement: fcac225_Supplementary_Data [file fcac225_Supplementary_Data.pdf]

Supplementary Figure 1. Graphical flowchart of patients' selection process

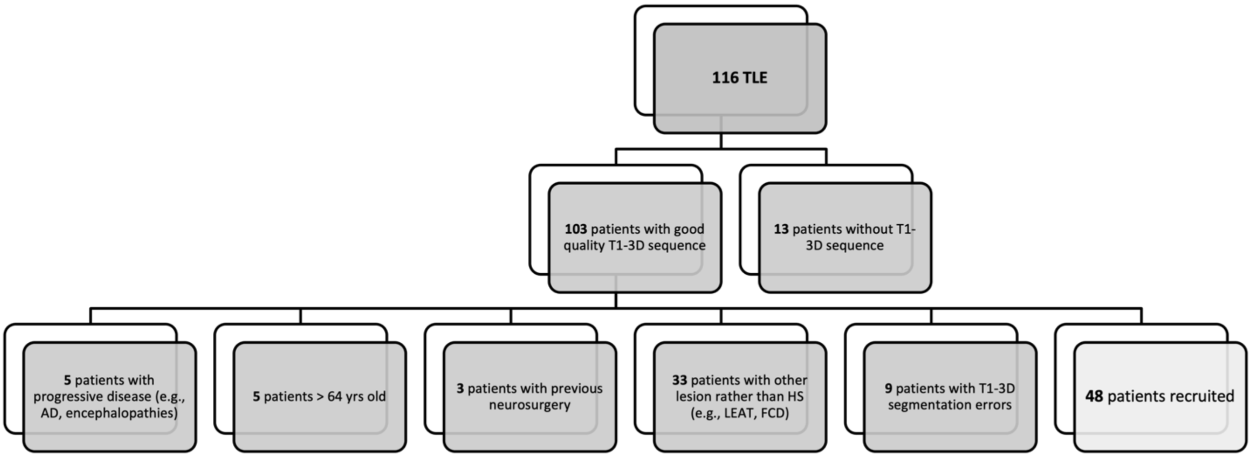

AD: Alzheimer Dementia, HS: hippocampal sclerosis, FCD: focal cortical dysplasia, LEAT: long-term epilepsy-associated tumors

Supplementary Table 1. MRI acquisition parameters

Instrumental Manufacture	Tesla	Slice	Slice thickness (mm)	TR - TE (ms)
**Philips Ingenia**	3			
T1-3D		170	1x1x1 mm	9,90 – 4,60
FLAIR-3D		300	1,12 mm	4800 – 305
Coronal T2		40	2,5 mm	3200 – 80
**GE Signa Architect**	3			
T1-3D		178	1x1x1 mm	7,8 – 3,1
FLAIR-3D		148	1,2 mm	6000 – 119
Coronal T2		60	2 mm	9600 – 90

TR: repetition time, TE: echo time

Supplementary Table 2. Independent sample t-test to assess the volumetric differences in the whole amygdala across the two different scanners in each patients' group after ComBat harmonization.

		GE	Philips	t	p-value	Cohen's D
**TLE-MRIneg**	*Left Whole Amygdala*	-0.116 (0.91)	0.363 (0.66)	-1.567	.131	-.649
	Right Whole Amygdala	-0.314 (0.80)	0.199 (1.13)	-1.305	.205	-.541
**TLE-HS**	*Left Whole Amygdala*	-0.828 (1.31)	-0.521 (1.01)	-.650	.523	-.269
	Right Whole Amygdala	-0.520 (0.63)	-0.251 (0.62)	-1.041	.309	-.431
**Extra-TLE**	*Left Whole Amygdala*	0.348 (0.95)	0.437 (1.24)	-.122	.904	-.091
	Right Whole Amygdala	0.248 (1.21)	-0.032 (1.12)	.314	.757	.234
**HC**	*Left Whole Amygdala*	-0.460 (0.55)	0.092 (1.05)	-1.131	.268	-.554
	Right Whole Amygdala	0.276 (0.32)	-0.055 (1.08)	.669	.509	.328

Z-scores are presented in means; Standard Deviations (SDs) are presented in the parentheses. t: independent sample t-test value.

Supplementary Table 3. Morphometric comparison of hippocampus subfields between patients' groups and HC

		TLE-MRIneg	TLE-HS	Extra-TLE	HC	F	p-value	Pairwise comparison[#]
Ipsilateral	Whole hippocampus	3463.050 (343.20)*	3033.712 (555.50)*	3579.647 (420.42)*	3594.078 (372.24)*	17.283	<.001***	TLE-HS < HC (p<.001)<br>TLE-HS < TLE-MRIneg (p<.001)<br>TLE-HS < Extra-TLE (p<.001)
	Hippocampal body	1161.882 (111.25)*	1048.308 (183.80)*	1204.401 (135.26)*	1230.988 (146.31)*	16.197	<.001***	TLE-HS < HC (p<.001)<br>TLE-HS < TLE-MRIneg (p<.001)<br>TLE-HS < Extra-TLE (p<.001)
	Hippocampal head	1745.404 (197.13)*	1479.709 (312.54)*	1799.072 (265.42)*	1794.492 (186.78)*	15.254	<.001***	TLE-HS < HC (p<.001)<br>TLE-HS < TLE-MRIneg (p<.001)<br>TLE-HS < Extra-TLE (p<.001)
	Hippocampal tail	556.375 (89.54)*	505.526 (91.23)*	577.447 (71.17)*	567.296 (81.54)*	9.108	<.001***	TLE-HS < HC (p<.001)<br>TLE-HS < TLE-MRIneg (p<.001)<br>TLE-HS < Extra-TLE (p<.001)
	Hippocampal fissure	145.113 (25.29)	142.137 (26.97)	139.996 (27.06)	145.298 (24.45)	1.393	.250	
	Subiculum	218.172 (24.25)	197.217 (40.19)	225.169 (28.56)	224.015 (28.04)	12.043	<.001***	TLE-HS < HC (p<.001)<br>TLE-HS < TLE-MRIneg (p<.001)<br>TLE-HS < Extra-TLE (p<.001)
	Presubiculum	155.359 (14.49)	137.027 (26.86)	157.822 (20.69)	154.126 (17.26)	11.393	<.001***	TLE-HS < HC (p<.001)<br>TLE-HS < TLE-MRIneg (p<.001)<br>TLE-HS < Extra-TLE (p<.001)
	Parasubiculum	65.022 (8.43)	59.848 (12.00)	70.885 (15.04)	65.996 (12.26)	2.669	.052	
	CA1	326.401 (38.92)	275.978 (59.86)	334.076 (46.22)	334.154 (37.95)	15.591	<.001***	TLE-HS < HC (p<.001)<br>TLE-HS < TLE-MRIneg (p<.001)<br>TLE-HS < Extra-TLE (p<.001)
	CA2/3	101.783 (15.41)	86.826 (17.26)	106.662 (19.16)	111.120 (16.64)	14.199	<.001***	TLE-HS < HC (p<.001)<br>TLE-HS < TLE-MRIneg (p<.001)<br>TLE-HS < Extra-TLE (p<.001)
	CA4	121.049 (14.04)	103.181 (21.86)	127.633 (18.01)	132.799 (15.47)	21.466	<.001***	TLE-HS < HC (p<.001)<br>TLE-HS < TLE-MRIneg (p<.001)<br>TLE-HS < Extra-TLE (p<.001)
	Molecular layer	281.993 (29.26)	241.893 (48.12)	290.766 (35.99)	293.879 (31.29)	18.602	<.001***	TLE-HS < HC (p<.001)<br>TLE-HS < TLE-MRIneg (p<.001)<br>TLE-HS < Extra-TLE (p<.001)
	GC-ML-GD	141.505 (16.03)	120.818 (26.01)	149.477 (21.41)	154.102 (16.71)	19.802	<.001***	TLE-HS < HC (p<.001)<br>TLE-HS < TLE-MRIneg (p<.001)<br>TLE-HS < Extra-TLE (p<.001)
	Fimbria	87.378 (16.07)	85.087 (22.14)	87.774 (19.61)	84.365 (18.23)	.391	.760	
	HATA	62.795 (10.96)	57.050 (11.93)	63.272 (12.29)	65.198 (9.77)	1.989	.121	
Contralateral	Whole hippocampus	3572.610 (440.79)	3119.425 (535.69)	3631.583 (386.61)	3554.490 (602.05)	1.958	.126	
	Hippocampal body	1179.300 (141.65)	1059.276 (200.88)	1192.835 (125.62)	1195.555 (185.88)	1.324	.271	
	Hippocampal head	1823.969 (265.36)	1564.629 (293.34)	1845.373 (236.66)	1789.566 (325.72)	2.335	.079	
	Hippocampal tail	569.773 (81.77)	496.691 (70.87)	592.852 (86.91)	568.312 (121.16)	1.157	.331	
	Hippocampal fissure	151.541 (35.48)	148.378 (26.76)	147.475 (27.00)	146.612 (26.50)	1.013	.391	
	Subiculum	214.356 (31.86)	193.413 (33.96)	215.835 (20.84)	215.282 (35.09)	.440	.725	
	Presubiculum	149.772 (19.15)	134.392 (30.12)	149.964 (18.67)	148.505 (25.54)	.288	.834	
	Parasubiculum	65.239 (10.97)	61.252 (14.86)	67.680 (12.16)	64.777 (14.58)	.269	.848	

CA1	348.016 (51.43)	294.517 (54.67)	349.679 (47.55)	338.639 (63.64)	2.474	.067	
CA2/3	111.806 (17.88)	97.688 (21.20)	116.032 (17.97)	111.782 (21.31)	2.226	.091	
CA4	127.840 (18.41)	109.281 (24.91)	131.024 (15.25)	130.026 (23.23)	3.454	.020*	TLE-HS < HC (p=.019)
Molecular layer	291.484 (37.60)	252.291 (46.30)	294.514 (33.45)	289.725 (50.65)	2.186	.095	
GC-ML-GD	148.952 (21.07)	127.420 (28.54)	153.289 (18.55)	150.801 (26.71)	3.122	.030*	TLE-HS < HC (p=.024)
Fimbria	87.019 (17.17)	81.620 (28.09)	82.659 (16.59)	84.754 (23.39)	.818	.487	
HATA	67.476 (13.07)	62.241 (10.77)	67.993 (11.51)	65.054 (13.87)	1.231	.303	

Data (in mm^3) are presented in means; Standard Deviations (SDs) are presented in the parentheses. Age, gender, and eTIV as covariates. F: MANCOVA's F-test value. *: p<.05, **: p<.01, ***: p<.001. $^{\#}$: significant p-value of pairwise comparisons between groups using post-hoc Bonferroni correction method (p < 0.05). GC-ML-GD: granule cell and molecular layer of the dentate gyrus, HATA: hippocampal-amygdala transition area.

Supplementary Table 4. Morphometric comparison of thalamus nuclei between patients' groups and HC

		TLE-MRIneg	TLE-HS	Extra-TLE	HC	F	p-value	Pairwise comparison[#]
Ipsilateral	Whole thalamus	6033.753 (645.17)	5622.358 (922.84)	6176.902 (1255.36)	6391.267 (1643.13)	1.846	.144	
	Anteroventral	116.675 (28.30)	89.434 (35.01)	121.368 (39.87)	126.567 (79.38)	2.639	.054	
	Laterodorsal	24.141 (8.84)	16.896 (7.63)	24.095 (11.35)	23.704 (15.54)	2.394	.074	
	Lateral posterior	107.824 (19.82)	102.656 (25.79)	114.214 (30.13)	114.824 (42.09)	.438	.726	
	Ventral anterior	372.307 (79.86)	337.315 (52.76)	377.727 (93.64)	372.751 (120.66)	.644	.589	
	Ventral anterior magnocellular	29.836 (7.19)	26.632 (4.82)	30.206 (7.52)	29.647 (9.67)	.921	.434	
	Ventral lateral anterior	577.197 (88.06)	547.983 (96.32)	587.664 (105.13)	600.412 (165.86)	.689	.561	
	Ventral lateral posterior	747.410 (89.07)	727.043 (108.46)	762.054 (115.33)	787.139 (163.08)	.908	.440	
	Ventral posterolateral	773.265 (77.48)	769.542 (98.24)	762.312 (129.78)	825.755 (134.87)	2.074	.109	
	Ventromedial	17.784 (2.06)	17.634 (2.21)	17.610 (3.11)	18.648 (2.76)	1.152	.333	
	Central medial	58.667 (16.39)	46.969 (13.62)	60.923 (18.32)	64.323 (32.84)	2.291	.083	
	Central lateral	29.344 (8.63)	24.156 (9.51)	31.797 (18.26)	31.614 (24.20)	1.256	.294	
	Paracentral	3.324 (0.62)	2.957 (0.71)	3.297 (0.68)	3.454 (0.92)	2.128	.102	
	Centromedian	231.366 (31.35)	226.507 (33.44)	234.739 (48.68)	246.221 (56.66)	1.131	.341	
	Parafascicular	51.989 (9.04)	53.522 (9.51)	52.578 (11.57)	55.778 (16.18)	1.203	.313	
	Paratenial	6.272 (1.07)	5.896 (1.25)	6.273 (1.63)	6.726 (2.67)	.724	.540	
	Reuniens (medial ventral)	9.939 (4.64)	7.878 (3.69)	11.474 (4.98)	12.137 (8.15)	1.880	.138	
	Mediodorsal medial magnocellular	714.387 (92.94)*	646.591 (89.00)*	743.365 (110.23)*	722.397 (91.75)*	4.174	.008**	TLE-HS < HC (p=.024)<br>TLE-HS < TLE-MRIneg (p=.025)<br>TLE-HS < Extra-TLE (p=.038)
	Mediodorsal lateral parvocellular	259.271 (31.51)	236.096 (30.18)	269.576 (40.09)	258.779 (41.46)	2.935	.038*	
	Limitans (suprageniculate)	16.567 (9.79)	20.111 (12.26)	21.468 (14.14)	21.397 (28.50)	.356	.785	
	Lateral geniculate	187.354 (28.04)	176.353 (26.86)	189.932 (46.00)	191.218 (47.47)	.723	.541	
	Medial geniculate	124.831 (26.69)	101.851 (27.93)	121.763 (38.84)	130.773 (60.30)	1.823	.148	
	Whole Pulvinar	390.691 (41.49)	365.274 (72.34)	409.433 (119.19)	434.700 (155.30)	2.366	.076	
	Pulvinar anterior	206.230 (21.05)	190.848 (32.52)*	214.639 (44.43)	222.571 (52.82)*	4.011	.010**	TLE-HS < HC (p=.005)
	Pulvinar medial	994.687 (120.98)	913.988 (192.28)	1072.069 (345.41)	1124.467 (425.90)	2.469	.067	
	Pulvinar lateral	168.116 (29.32)	162.324 (45.76)	154.355 (34.90)	183.251 (77.16)	2.623	.055	
	Pulvinar inferior	193.730 (47.71)	193.937 (53.46)	196.671 (70.21)	208.510 (70.91)	1.041	.378	
Contralateral	Whole thalamus	5963.872 (597.03)	5816.397 (806.59)	6204.734 (1293.21)	6354.943 (1221.14)	1.190	.318	

Anteroventral	113.550 (30.61)	97.122 (34.89)	121.255 (44.45)	120.503 (42.25)	1.549	.207
Laterodorsal	20.842 (9.39)	17.901 (8.61)	21.394 (12.50)	21.706 (13.63)	.831	.480
Lateral posterior	102.216 (22.31)	101.572 (20.59)	108.441 (30.82)	104.295 (27.67)	.572	.635
Ventral anterior	361.867 (53.77)	342.695 (45.99)	378.841 (102.47)	359.444 (120.87)	.322	.809
Ventral anterior magnocellular	29.435 (5.31)	27.193 (4.23)	29.997 (7.53)	30.153 (8.39)	.517	.671
Ventral lateral anterior	574.082 (64.42)	568.147 (76.86)	586.076 (122.61)	586.467 (99.29)	.175	.913
Ventral lateral posterior	744.540 (76.46)	747.156 (101.40)	758.578 (125.69)	767.008 (92.33)	.372	.774
Ventral posterolateral	750.409 (79.92)	769.660 (119.92)	783.899 (127.96)	802.308 (122.76)	1.316	.274
Ventromedial	17.671 (2.33)	17.982 (3.45)	17.542 (3.30)	18.138 (2.89)	.878	.456
Central medial	58.492 (13.89)	48.496 (10.40)	59.836 (20.17)	61.486 (19.46)	2.358	.077
Central lateral	26.815 (8.42)	24.130 (8.33)	29.980 (14.69)	30.116 (14.36)	1.334	.268
Paracentral	3.230 (0.51)	2.931 (0.49)	3.265 (0.70)	3.398 (0.43)	2.109	.105
Centromedian	225.529 (25.59)	230.467 (34.47)	231.514 (47.51)	231.518 (28.85)	.393	.758
Parafascicular	49.477 (5.83)	53.548 (8.89)	52.645 (12.80)	52.805 (6.83)	1.638	.186
Paratenial	6.030 (1.18)	5.707 (1.32)	6.283 (2.02)	6.317 (2.40)	.864	.463
Reuniens (medial ventral)	9.724 (4.75)	7.619 (3.19)	11.500 (5.33)	11.230 (6.48)	2.631	.055
Mediodorsal medial magnocellular	706.722 (79.37)	669.502 (76.98)	721.687 (90.36)	726.150 (63.37)	1.858	.142
Mediodorsal lateral parvocellular	257.944 (29.17)	247.979 (30.67)	260.932 (34.49)	261.286 (26.53)	.651	.584
Limitans (suprageniculat e)	14.731 (8.74)	18.911 (12.75)	20.750 (13.22)	19.259 (22.76)	.740	.531
Lateral geniculate	193.220 (32.08)	181.161 (44.04)	188.880 (42.96)	199.149 (54.72)	.489	.691
Medial geniculate	113.479 (26.85)	103.770 (26.10)	124.607 (41.56)	122.670 (54.59)	1.343	.265
Whole Pulvinar	393.342 (34.70)	381.813 (71.66)	433.647 (126.03)	452.332 (135.32)	2.023	.116
Pulvinar anterior	205.721 (15.94)	202.404 (24.37)	222.299 (44.81)	227.554 (28.35)	2.959	.036*
Pulvinar medial	989.486 (94.99)	944.802 (192.02)	1116.151 (339.83)	1142.826 (352.20)	2.601	.057
Pulvinar lateral	176.701 (42.34)	183.075 (44.47)	178.335 (56.08)	196.806 (59.13)	.477	.699
Pulvinar inferior	201.462 (35.72)	196.970 (52.29)	217.804 (82.57)	242.142 (120.65)	.837	.477

Data (in mm^3) are presented in means; Standard Deviations (SDs) are presented in the parentheses. Age, gender, and eTIV as covariates. F: MANCOVA's F-test value. *: $p<.05$, **: $p<.01$, ***: $p<.001$. #: significant p-value of pairwise comparisons between groups using post-hoc Bonferroni correction method ($p < 0.05$).

Supplementary Table 5. Independent sample t-test comparison of thalamus nuclei between TLE-HS and HC populations

		**TLE-HS**	**HC**	**t**	**p-value**	**Cohen's D**
Ipsilateral	Whole thalamus	5622.358 (922.84)	6391.267 (1643.13)	-2.181	.034*	-.597
	Anteroventral	89.434 (35.01)	126.567 (79.38)	-2.373	.021*	-.650
	Laterodorsal	16.896 (7.63)	23.704 (15.54)	-1.774	.082	-.486
	Lateral posterior	102.656 (25.79)	114.824 (42.09)	-0.792	.432	-.217
	Ventral anterior	337.315 (52.76)	372.751 (120.66)	-1.126	.265	-.308
	Ventral anterior magnocellular	26.632 (4.82)	29.647 (9.67)	-1.577	.121	-.432
	Ventral lateral anterior	547.983 (96.32)	600.412 (165.86)	-1.275	.208	-.349
	Ventral lateral posterior	727.043 (108.46)	787.139 (163.08)	-1.362	.179	-.373
	Ventral posterolateral	769.542 (98.24)	825.755 (134.87)	-1.456	.151	-.399
	Ventromedial	17.634 (2.21)	18.648 (2.76)	-1.145	.258	-.313
	Central medial	46.969 (13.62)	64.323 (32.84)	-2.622	.011*	-.718
	Central lateral	24.156 (9.51)	31.614 (24.20)	-1.557	.126	-.426
	Paracentral	2.957 (0.71)	3.454 (0.92)	-2.270	.027*	-.622
	Centromedian	226.507 (33.44)	246.221 (56.66)	-1.352	.182	-.370
	Parafascicular	53.522 (9.51)	55.778 (16.18)	-0.184	.855	-.050
	Paratenial	5.896 (1.25)	6.726 (2.67)	-1.162	.251	-.318
	Reuniens (medial ventral)	7.878 (3.69)	12.137 (8.15)	-2.308	.025*	-.632
	Mediodorsal medial magnocellular	646.591 (89.00)	722.397 (91.75)	-3.449	.001**	-.945
	Mediodorsal lateral parvocellular	236.096 (30.18)	258.779 (41.46)	-2.697	.009**	-.739
	Limitans (suprageniculate)	20.111 (12.26)	21.397 (28.50)	-0.135	.893	-.037
	Lateral geniculate	176.353 (26.86)	191.218 (47.47)	-1.584	.119	-.434
	Medial geniculate	101.851 (27.93)	130.773 (60.30)	-1.975	.054	-.541
	Whole Pulvinar	365.274 (72.34)	434.700 (155.30)	-2.341	.023*	-.641
	Pulvinar anterior	190.848 (32.52)	222.571 (52.82)	-3.388	.001**	-.928
	Pulvinar medial	913.988 (192.28)	1124.467 (425.90)	-2.471	.017*	-.677
	Pulvinar lateral	162.324 (45.76)	183.251 (77.16)	-1.642	.107	-.450
	Pulvinar inferior	193.937 (53.46)	208.510 (70.91)	-1.517	.135	-.415
Contralateral	Whole thalamus	5816.397 (806.59)	6354.943 (1221.14)	-1.748	.086	-.479
	Anteroventral	97.122 (34.89)	120.503 (42.25)	-2.008	.050	-.550
	Laterodorsal	17.901 (8.61)	21.706 (13.63)	-1.400	.167	-.383
	Lateral posterior	101.572 (20.59)	104.295 (27.67)	-0.985	.329	-.270
	Ventral anterior	342.695 (45.99)	359.444 (120.87)	-0.854	.397	-.234
	Ventral anterior magnocellular	27.193 (4.23)	30.153 (8.39)	-1.385	.172	-.379
	Ventral lateral anterior	568.147 (76.86)	586.467 (99.29)	-0.830	.410	-.227
	Ventral lateral posterior	747.156 (101.40)	767.008 (92.33)	-0.923	.360	-.253
	Ventral posterolateral	769.660 (119.92)	802.308 (122.76)	-1.274	.208	-.349
	Ventromedial	17.982 (3.45)	18.138 (2.89)	-0.435	.665	-.119
	Central medial	48.496 (10.40)	61.486 (19.46)	-2.670	.010*	-.731
	Central lateral	24.130 (8.33)	30.116 (14.36)	-1.687	.098	-.462
	Paracentral	2.931 (0.49)	3.398 (0.43)	-2.950	.005**	-.808
	Centromedian	230.467 (34.47)	231.518 (28.85)	-0.585	.561	-.160
	Parafascicular	53.548 (8.89)	52.805 (6.83)	0.154	.879	.042
	Paratenial	5.707 (1.32)	6.317 (2.40)	-1.390	.171	-.381
	Reuniens (medial ventral)	7.619 (3.19)	11.230 (6.48)	-2.566	.013*	-.703
	Mediodorsal medial magnocellular	669.502 (76.98)	726.150 (63.37)	-2.688	.010*	-.736
	Mediodorsal lateral parvocellular	247.979 (30.67)	261.286 (26.53)	-1.459	.151	-.400
	Limitans (suprageniculate)	18.911 (12.75)	19.259 (22.76)	-0.171	.865	-.047
	Lateral geniculate	181.161 (44.04)	199.149 (54.72)	-1.041	.303	-.285
	Medial geniculate	103.770 (26.10)	122.670 (54.59)	-1.774	.082	-.486
	Whole Pulvinar	381.813 (71.66)	452.332 (135.32)	-1.970	.054	-.539

Pulvinar anterior	202.404 (24.37)	227.554 (28.35)	-2.585	.013*	-.708
Pulvinar medial	944.802 (192.02)	1142.826 (352.20)	-2.222	.031*	-.609
Pulvinar lateral	183.075 (44.47)	196.806 (59.13)	-0.531	.598	-.145
Pulvinar inferior	196.970 (52.29)	242.142 (120.65)	-1.324	.191	-.363

Data (in mm^3) are presented in means; Standard Deviations (SDs) are presented in the parentheses. t: pair t-test value. *: p<.05, **: p<.01, ***: p<.001.

Supplementary Table 6. Asymmetry of the amygdala subnuclei in HC

	Left	Right	t	p-value	Cohen's D
Whole amygdala	1793.36 (206.08)	1802.04 (361.90)	0.000	1.000	0.000
Lateral nucleus	671.10 (76.95)	665.22 (130.45)	0.000	1.000	0.000
Basal nucleus	455.57 (58.10)	458.57 (92.44)	0.000	1.000	0.000
AB nucleus	276.48 (33.86)	280.67 (62.05)	0.001	0.999	0.000
Paralaminar nucleus	51.30 (6.72)	51.18 (10.28)	0.000	1.000	0.000
Central nucleus	47.43 (7.96)	48.94 (15.44)	0.000	1.000	0.000
Medial nucleus	19.82 (4.80)	21.52 (8.11)	0.001	0.999	0.000
Cortical nucleus	26.69 (4.02)	27.41 (7.38)	-0.001	0.999	0.000
AAA	55.43 (7.82)	55.45 (10.57)	-0.001	1.000	0.000
CAT	189.54 (27.67)	193.07 (37.18)	0.000	1.000	0.000

Data (in mm^3) are presented in means; Standard Deviations (SDs) are presented in the parentheses. t: pair t-test value. *: p<.05, **: p<.01, ***: p<.001. AAA: anterior amygdaloid area, CAT: corticoamygdaloid transition area.

Supplementary Table 7. Subcortical gray matter volume, total gray matter volume, and total white matter volume across groups

	TLE-MRIneg	TLE-HS	Extra-TLE	HC
Total GMV	609633.056 (±64655.42)	592671.643 (±65651.62)	649458.956 (±79557.82)	618871.172 (±62253.99)
Subcortical GMV	56122.958 (+4995.66)	52865.417 (+6153.28)	56933.850 (+6194.57)	57755.633 (+6332.06)
WMV	434526.053 (+46842.76)	443144.776 (+60019.35)	462897.068 (+61169.56)	474563.595 (+63910.73)

Data (in mm^3) are presented in means, Standard Deviations (SDs) are presented in the parentheses. GMV: gray matter volume, WMV: white matter volume.
